# Selective optogenetic control of G_q_ signaling using human Neuropsin

**DOI:** 10.1038/s41467-022-29265-w

**Published:** 2022-04-01

**Authors:** Ahmed Wagdi, Daniela Malan, Udhayabhaskar Sathyanarayanan, Janosch S. Beauchamp, Markus Vogt, David Zipf, Thomas Beiert, Berivan Mansuroglu, Vanessa Dusend, Mark Meininghaus, Linn Schneider, Bernd Kalthof, J. Simon Wiegert, Gabriele M. König, Evi Kostenis, Robert Patejdl, Philipp Sasse, Tobias Bruegmann

**Affiliations:** 1 Institute for Cardiovascular Physiology, University Medical Center Göttingen, Göttingen, Germany; 2 German Center for Cardiovascular Research (DZHK), Partner site Göttingen, Göttingen, Germany; 3 Institute of Physiology I, Medical Faculty, University of Bonn, Bonn, Germany; 4 Department of Internal Medicine II, University Hospital Bonn, University of Bonn, Bonn, Germany; 5 Research Training Group 1873, University of Bonn, Bonn, Germany; 6 Bayer AG, Research & Development, Pharmaceuticals, 42096 Wuppertal, Germany; 7 Center for Molecular Neurobiology Hamburg, University Medical Center Hamburg-Eppendorf, Hamburg, Germany; 8 Molecular, Cellular and Pharmacobiology Section, Institute of Pharmaceutical Biology, University of Bonn, Bonn, Germany; 9 Oscar-Langendorff Institute of Physiology, Rostock University Medical Center, Rostock, Germany; 10 Cluster of Excellence “Multiscale Bioimaging: from Molecular Machines to Networks of Excitable Cells” (MBExC), University of Göttingen, Göttingen, Germany; 11 Present Address: Department of Cardiology and Pulmonology, University Medical Center Göttingen, Georg August University of Göttingen, Göttingen, Germany

**Keywords:** Drug development, Arrhythmias, Protein design, Optogenetics

## Abstract

G_q_ proteins are universally important for signal transduction in mammalian cells. The underlying kinetics and transformation from extracellular stimuli into intracellular signaling, however could not be investigated in detail so far. Here we present the human Neuropsin (hOPN5) for specific and repetitive manipulation of G_q_ signaling in vitro and in vivo with high spatio-temporal resolution. Properties and G protein specificity of hOPN5 are characterized by UV light induced IP_3_ generation, Ca^2+^ transients and inhibition of G_IRK_ channel activity in HEK cells. In adult hearts from a transgenic animal model, light increases the spontaneous beating rate. In addition, we demonstrate light induced contractions in the small intestine, which are not detectable after pharmacological G_q_ protein block. All-optical high-throughput screening for TRPC6 inhibitors is more specific and sensitive than conventional pharmacological screening. Thus, we demonstrate specific G_q_ signaling of hOPN5 and unveil its potential for optogenetic applications.

## Abstract (web summary)

Gq proteins are one of four major classes of G proteins; optogenetic receptors for selective and repetitive activation of Gq proteins with fast kinetics are lacking. Here the authors report UV light-dependent Gq signalling using human Neuropsin (hOPN5) and demonstrate its potential as an optogenetic tool.

## Introduction

G protein coupled receptors (GPCRs) are the largest class of cell surface receptors and transform extracellular information into intracellular signaling for the regulation of enzymes, ion channels, transporters, and other components of the cellular machinery^1–5^. G_q_ proteins are one of four major classes of G proteins^2^ which can be subdivided according to the respective α subunits G_q_, G_11_, G_14_, and G_15/16_^3,6,7^. Once activated by the GPCR, G_q_ α subunits trigger phospholipase Cβ (PLC-β) activity, which hydrolyses phosphatidylinositol-4,5-bisphosphate (PIP2) into diacylglycerol (DAG) and inositol 1,4,5 trisphosphate (IP_3_). DAG activates the protein kinase C and transient receptor potential channels (TRPC)^8^ whereas IP_3_ initiates the release of Ca^2+^ ions from the endoplasmic reticulum^2,6,9^. The two-second messengers together with various other non-canonical pathways^6,9,10^ control a plethora of different aspects of cellular function. These include contraction of smooth muscle cells, regulation of contractility in cardiomyocytes^8^, gene expression, posttranslational modification, and various other mechanisms in almost every cell type^9^. Since G_q_ signaling plays a major role for the physiological adaption of cellular function it is also involved in the pathogenesis of a myriad of diseases^10^ being key for platelet aggregation^11^, synaptic plasticity^11^, autoimmunity^12^, cancerogenesis^13,14^, physiological as well as pathological cardiac growth and the development of heart failure^8,11,15^.

Over the past century, a lot of “binary” knowledge was gathered about mechanisms leading to activation or inactivation of GPCRs by drugs, hormones, transmitters, ions as well as physical stimuli, e.g. light, stretch, or depolarization^4,16,17^. However, the temporal encoding, subcellular compartmentalization, as well as microdomain discrimination is less understood since the tools to investigate G_q_ signaling have been restricted to pharmacological or genetic approaches both lacking spatial and temporal precision^7^. Optogenetics is a technique that allows to control cellular behavior with light after expressing light-sensitive proteins in cell-types of interest. The advantages of optogenetic stimulation are unprecedented temporal and spatial precision, cell-type-specific manipulations, and subcellular targeting. Thus optogenetics has been used to investigate GPCR signaling in cells^18–20^, the heart^21,22^, the eye^23–25^, and the brain^7,19,26–29^. Ideally, optogenetic GPCRs should be selective for only one of the four major G protein classes. However, specific non-promiscuous optogenetic receptors have only been described for G_s_ proteins (JellyfishOpsin)^21,30^ and G_i/o_ proteins (Rhodopsin/Coneopsin)^27^, whereas optogenetic receptors for selective and repetitive activation of only G_q_ proteins with fast kinetics are missing. For instance, Melanopsin (OPN4) has been shown to activate G_q_ proteins in some cell-types^19,22,29,31,32^ but has a pronounced promiscuity since it can also activate G_i/o_ proteins^19,31^.

The so-called Opto-α1-Adrenoreceptor, a chimera of the light-sensitive Rhodopsin and the intracellular parts of the α1-Adrenoreceptor thought to be responsible for G_q_ protein binding, has been reported^33^. However, Opto-α1-Adrenoreceptor has ever since only been sporadically used due to problems like bleaching leading to weak and non-repeatable effects.

The mammalian neuronal tissue opsin Neuropsin (OPN5) has been found in many species from fishes to birds and humans and is described as a bi-stable photoreceptor that can be activated by UV light and deactivated with red-shifted light (>470 nm)^34–39^. In mammals, OPN5 expression has been detected in the brain, eye, skin, and testis tissue as well as the outer ear^36,39,40^. Only recently the physiological role of OPN5 has been linked to the photoentrainment of the retina^41^ and melanocytes^42^ as well as to light-induced thermogenesis controlled via the preoptic area of the hypothalamus^43^. The underlying pathway, however, is still unclear since OPN5 activation of G_i/o_ as well as G_q_ proteins has been suggested^35,44^, but a systematic analysis of its specificity towards the individual G protein classes has not been performed yet.

Here, we characterize in detail the functional behavior of human OPN5 (hOPN5) and prove that hOPN5 enables specific and repetitive activation of G_q_ signaling without promiscuous stimulation of G_i/o_ proteins in HEK cells, cardiomyocytes, the adult mouse heart as well as smooth muscle cells of small intestine, bladder, and uterus. Furthermore, we establish a biotechnological application using hOPN5 stimulation in an all-optical high throughput screening (HTS) assay for TRPC6 inhibitors and show the major advantage of light-activated G_q_ signaling by OPN5 in handling, sensitivity, and specificity over conventional compound screening using pharmacological stimulation.

## Results

### hOPN5 activates G_q_ signaling

**Fig. 1:**
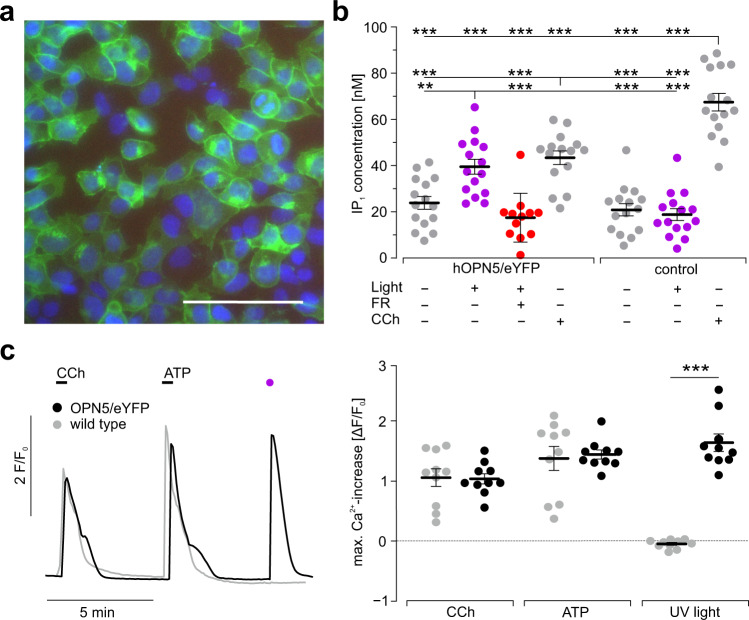
hOPN5 activates G_q_ proteins in HEK cells. **a** Transgenic HEK cells expressing hOPN5 in fusion with eYFP (green), nuclei stained with DAPI (blue), bar = 100 µm; **b** IP_1_ levels in hOPN5/eYFP and wild-type HEK cells without stimulation (gray) and after UV light stimulation (50 µW/mm^2^, 1 s every 5 min for 30 min), without (violet) or with FR900359 (1 µM, red) to block G_q_ proteins and after CCh application (300 µM, 30 min, gray). Each dot represents the result from one well. Statistical analysis with one-way ANOVA and Tukey’s multiple comparisons post-test (p(WT vs. WT light): 0.99; p(WT vs. hOPN5): 0.99; p(WT vs. hOPN5 FR): 0.99; p(WT light vs. hOPN5): 0.89; p(WT light vs. hOPN5 FR): 0.99; p(hOPN5 vs. hOPN5 FR): 0.79; p(hOPN5 CCh vs. hOPN5 light): 0.97). **c** Representative Ca^2+^ transients (left) induced by UV light (violet, 1 mW/mm^2^, 1 s) and CCh (2 mM) or ATP (1.5 mM) in hOPN5/eYFP (black) and wild-type (gray) HEK cells and statistical comparison of the maximal Ca^2+^ transient amplitudes (right, *n* = 10, each dot is the average of one region of interest consisting of at least 100 cells) with two-way ANOVA test with Sidak’s multiple comparison test for the difference between hOPN5 and control HEK cells (p(CCh) and p(ATP) = 0.99).

To characterize the downstream effects of hOPN5 activation, we generated a stable HEK cell line expressing hOPN5 in fusion with eYFP at the C-terminus, which showed cell membrane localization (Fig. 1a). Activation of the G_q_ signaling cascade was determined by measuring light and agonist-induced accumulation of IP_1_, which is the degradation product of IP_3_. Illumination with UV light (385 nm) elevated IP_1_ levels in transgenic hOPN5 HEK cells but not in wild-type cells (Fig. 1b). This effect was completely abolished by the G_q_ specific blocker FR900359^45^. Both, wild type and hOPN5 HEK cells showed elevated IP_1_ levels after pharmacological activation of the muscarinic receptor 3 (M3) by carbachol (CCh). Basal IP_1_ levels were not altered by hOPN5 expression itself excluding basal dark activity (Fig. 1b).

**Fig. 2:**
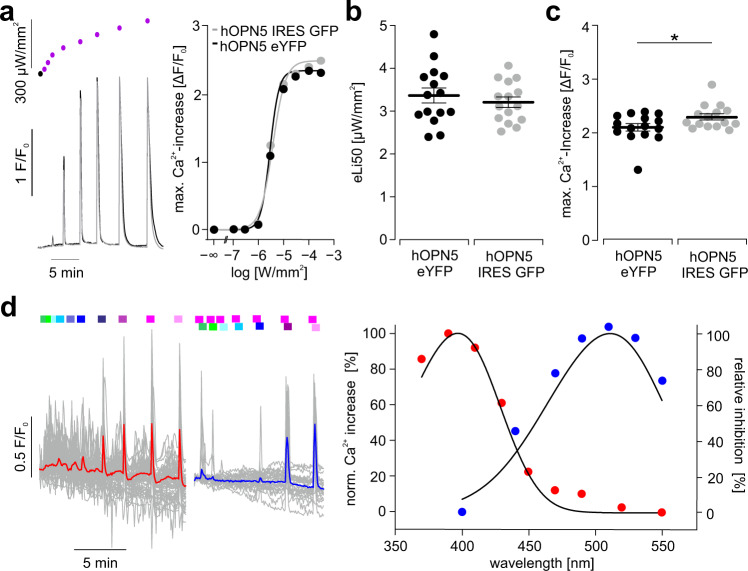
Functional determination of light sensitivity and wavelength specificity. **a** Representative Ca^2+^ transients after 100 ms long light pulses with increasing light intensity (control: black dot, violet dots from left: 0.1, 0.3, 1, 3, 10, 30, 100, and 300 µW/mm^2^, light intensities indicated with logarithmic scale) and hill fit to analyze the half maximal effective light intensity (eLi50, right) of the maximal Ca^2+^ transient amplitudes in HEK cells expressing hOPN5 either in fusion with eYFP (black) or separately from cytosolic GFP expression (gray). **b** Analysis of the eLi50 (*p* = 0.47) as well as **c**, the maximal Ca^2+^ transient amplitudes (*p* = 0.042) in both cell lines (unpaired, two-sided Student’s *t* test), one dot represents the average of one coverslip. **d** Representative Ca^2+^ transients (left, 55 and 26 individual cells in gray, average in red and blue) induced with different wavelengths (red, 30 ms, from left in nm: 550, 530, 510, 490, 470, 450, 430, 410, 390, 370) and blue after activating with UV light (390 nm, 30 ms) followed by deactivation with different wavelengths (300 ms, from left in nm: 550, 530, 510, 490, 470, 440, 400) and analysis of wavelength specificity (right) for activation (red) and deactivation (blue, calculated as % of inhibition) fitted by Govardovskii equation (black, *R*^2^  =  0.98 and 0.93). Data are presented as mean values ± SEM. *p < 0.05, **p <  0.01, ***p  <  0.001.

To quantify the activation of G_q_ signaling by hOPN5 in more detail, we used Ca^2+^ imaging with X-Rhod-1 AM, a red-shifted monometric Ca^2+^ indicator to avoid hOPN5 activation or deactivation by the imaging light (558–586 nm, 50 ms excitation, 0.13 mW/mm^2^). Illumination with UV light (385 nm, 1 mW/mm^2^) consistently induced Ca^2+^ transients in hOPN5 HEK cells but not in wild-type controls (Fig. 1c). Light-induced Ca^2+^ transients were of similar peak amplitude compared to pharmacological stimulation of the endogenous G_q_ coupled M3 receptors with CCh or P2Y receptors with ATP (Fig. 1c) suggesting similar coupling efficacy. Importantly, the pharmacologically induced Ca^2+^ transients were not different in hOPN5 expressing and wild-type control cells (Fig. 1c) proving that hOPN5 overexpression did not influence endogenous G_q_ protein signaling. To determine the light sensitivity, we applied brief (100 ms) UV light pulses with increasing light intensities and found that even low light levels of 1 µW/mm^2^ were able to induce Ca^2+^ transients (Fig. 2a and Supplementary Video 1). Similar to receptor pharmacology, the amplitude of Ca^2+^ transients could be gradually controlled with a sigmoidal dependence on log[light intensity] and a half maximal light intensity (eLi50) of ~3 µW/mm^2^ (Fig. 2a, b). To exclude side effects by eYFP fusion to OPN5, we generated an additional hOPN5 HEK cell line with cytosolic GFP expression using an internal ribosome entry sequence (IRES). This cell line showed slightly higher light-induced Ca^2+^ transients (Fig. 2a, c) but identical eLi50 light sensitivity (Fig. 2a, b) compared to the OPN5/eYFP fusion cell line. The spectral response of hOPN5 was determined using light pulses ranging from 370 to 550 nm (Fig. 2d). We found maximal activation at 406 ± 2.4 nm (*N* = 11). Because hOPN5 is suggested to be a bi-stable opsin^34,35,39^, we tested its deactivation by a second light pulse of longer wavelengths (400 to 550 nm, 300 ms, >1 mW/mm^2^) directly applied (~4 ms delay) after supramaximal stimulation with 385 nm. This led to inhibition of Ca^2+^ transients and the effect was maximal at 511 ± 1.7 nm (Fig. 2d, *N* = 10). Next, we determined the desensitization of OPN5 mediated Ca^2+^ transients. When we applied paired 1, 3, and 10 s long light pulses starting directly after the first light-induced Ca^2+^ transient, we found increasing desensitization (Supplementary Fig. 1) but a fast recovery resulting in >90% effectivity ~250 s after a 3 s light pulse.

Altogether, this data demonstrates that hOPN5 activates the G_q_ signaling cascade upon illumination which results in IP_3_ production as well as Ca^2+^ transients. Activation is most effective with UV light and can be repetitively induced. However, this does not exclude promiscuity of hOPN5 and hidden activation of G_i_ proteins yet.

### hOPN5 does not activate G_i_ proteins

**Fig. 3:**
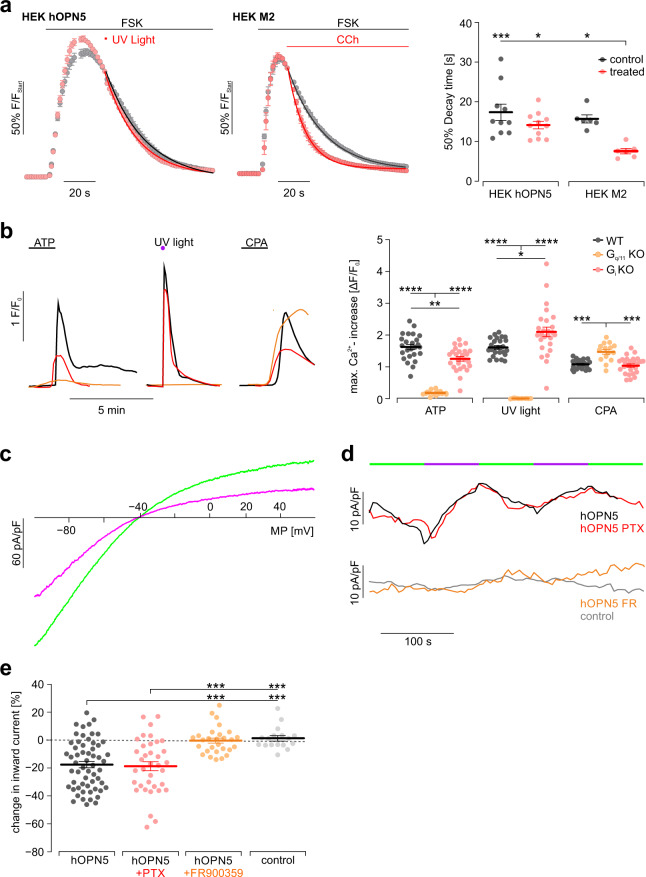
hOPN5 does not activate G_i_ proteins in HEK cells. **a** Averaged time courses (left, average of 10, 11, and 6 individual wells for hOPN5 and M2, respectively) of cAMP levels analysed by GloSensor luminescence after adenylate cyclase stimulation with Forskolin (10 µM, FSK) and normalized to application of UV light (F_Start,_ 500 ms, 110 µW/mm^2^) or CCh (100 µM) in HEK cells expressing hOPN5 or the muscarine ACh receptor 2 (M2). Statistical analysis (right) of 50% decay time analyzed by exponential fit of each well individually (each dot represents one well) with ordinary one-way ANOVA with Tukey’s multiple comparison test (p(hOPN5 control vs. hOPN5 light): 0.32; p(hOPN5 control vs. M2 control): 0.87; p(M2 control vs. M2 CCh): 0.012; p(hOPN5 light vs. M2 CCh): 0.024). **b** Representative traces (left) and aggregated data (right) of Ca^2+^ transients induced by 1.5 mM ATP, UV light (1 mW/mm^2^, 1 s) and 50 µM cyclopiazonic acid (CPA) in HEK WT cells (black), in HEK G_i_ KO cells (red) and in HEK G_q/11_ KO cells (orange). Statistical analysis with a two-way ANOVA repeated measurements test. Indicated p values are from a Tukey’s multiple comparison test for the varying effects of the stimulations (*N* = 15 for G_q/11_ KO cells, *N* = 26 for G_i_ KO cells, and *N* = 25 for HEK WT cells; *p* = 0.76 for HEK WT versus G_i_ KO cells in CPA treatment). **c** Representative current-voltage ramps (outward currents in positive direction) of hOPN5/GIRK HEK cells during UV (385 nm, 1 mW/mm^2^, 75 s, violet) and green (500–600 nm, 14 mW/mm^2^, 75 s, green) illumination. **d** Time course of inward currents (in negative direction) at −80 mV during UV (violet bars) and green illumination (green bars). **e** UV light induced change in inward current at −80 mV (each dot represents one individual cell) in control and hOPN5 cells without and with PTX or FR900359 treatment. Statistics with a non-parametric ANOVA test with Tukey’s multiple comparison post-test. p(hOPN5 vs hOPN5 PTX) = 0.99; p(hOPN5 FR vs control) = 0.99. Data are presented as mean values ±SEM. *p <  0.05, **p <  0.01, ***p  <  0.001. ****p  <  0.0001.

To investigate the ability of hOPN5 to additionally activate G_i_ proteins, we measured changes in cAMP levels which was used before to propose G_i_ protein activation by hOPN5^39,44^. In contrast to previous reports, we could not detect any light effect on cAMP levels in hOPN5 expressing HEK cells despite using supramaximal UV light intensities for Ca^2+^ transients and clear cAMP reduction in control experiments with stimulation of the G_i_ coupled muscarinergic receptor 2 (Fig. 3a). Next, we analyzed Ca^2+^ transients in hOPN5 HEK cells lacking either G_i_ or G_q/11_ protein expression^45,46^. Light-induced Ca^2+^ transients were fully abolished in G_q/11_ knockout (KO) cells whereas ATP still evoked small Ca^2+^ responses (Fig. 3b). Furthermore, UV light-induced Ca^2+^ transients were even slightly higher in G_i_ KO cells compared to wild-type HEK cells. Importantly all three cell lines had intact endoplasmatic Ca^2+^ storage and release because blocking the SERCA by cyclopiazonic acid (CPA, Fig. 3b) elevated cytosolic Ca^2+^ levels. In order to prove that UV illumination of hOPN5/eYFP is not activating G_i_ signaling, we took advantage of the GIRK channel assay with its high level of sensitivity to reveal even small G_i_ protein activation and which had already been used to show the promiscuous nature of Melanopsin activating also G_i_ signaling^19,47^. Specifically it was shown that illumination of Melanopsin in HEK cells overexpressing GIRK channels 1 and 2 leads to increased GIRK currents by the G_i_ βγ subunit complex^19,47^. In contrast, specific activation of the G_q_ signaling cascade only would inhibit GIRK channels via PLC-dependent PIP2 depletion as well as by protein kinase Cδ mediated phosphorylation^48^. Thus, the GIRK assay is ideally suited to determine the promiscuity of a certain GPCR to activate either G_i_ or G_q_ proteins or both. Importantly, UV illumination led to a clear reduction of GIRK currents in HEK cells expressing hOPN5/eYFP and GIRK1/2 but not in control cells only expressing GIRK 1/2 (Fig. 3c–e). The UV light-induced GIRK inhibition was neither smaller nor larger after incubating the cells with the specific G_i_ protein blocker Pertussis toxin and only abolished and not reversed after the G_q_ blocker FR900359 (Fig. 3d-e). Thus, we conclude that hOPN5 is specific for G_q_ proteins and can exclude any activation of G_i_ proteins in HEK cells.

### All-optical high throughput screening with hOPN5

**Fig. 4:**
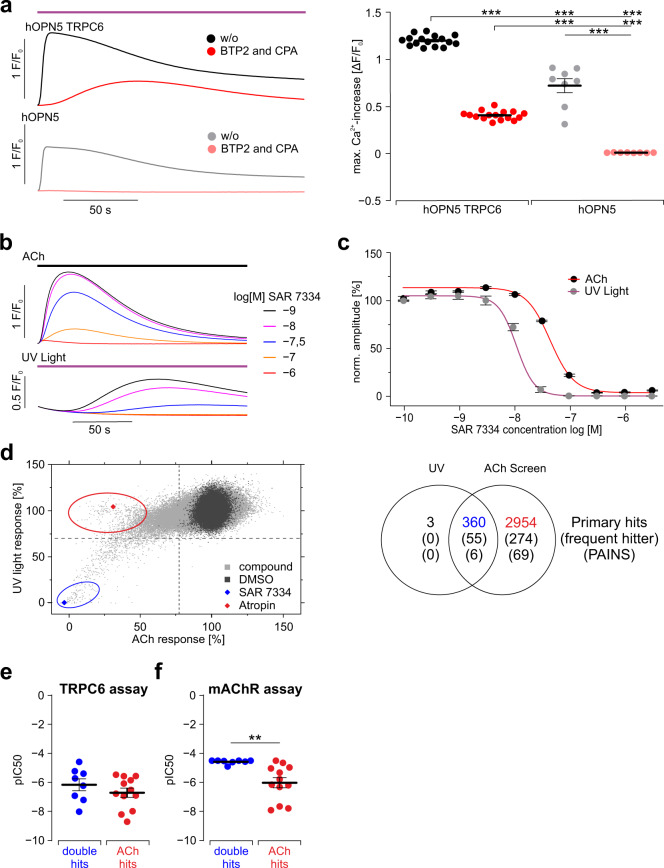
All-optical HTS improves TRPC6 inhibitor screening. **a** Representative Fluo-8 Ca^2+^ transients (left) and statistical analysis (right) of the maximal Ca^2+^ transient amplitudes in hOPN5/TRPC6 HEK cells and hOPN5 HEK cells during UV light stimulation (violet bar, 370 nm, 5 µW/mm^2^). Ca^2+^ release from internal stores was blocked with BTP2 (2 µM) and CPA (10 µM) in a subset of experiments (red/light red). Dots represent results from individual wells and statistical analysis was performed with ordinary one-way ANOVA test with Tukey’s multiple comparison post-test (all *p*-values < 0.0001). **b**, **c** Dose response-analysis of TRPC6 blocking effect of SAR 7334 for ACh mediated (100 µM) and UV light-induced (24 µW/mm^2^) Ca^2+^ transients. Representative UV light and ACh-induced Ca^2+^ transients under BTP2 and CPA (average of a quadruplicate) in the presence of different concentrations of SAR 7334 (b). Dose response-analysis of the TRPC6 blocking effect with a Hill fit for ACh (red fit, black dots) and for UV light-induced (violet fit, gray dots) Ca^2+^ transients (**c**, *N* = 4). **d** Normalized effects of 218,064 test compounds (left, gray, at 6 µM) in the HTS on TRPC6-dependent Ca^2+^ transients induced by ACh or UV light with DMSO controls (black), atropine (red diamond, 6 µM) and SAR 7334 (blue diamond, 3 µM). **d** Comparison (right) of primary hits, “frequent hitter” and PAINS (right) found only in the all-optical HTS (black), only in ACh HTS (ACh hits, red) or in both screens (double hits, blue). **e**, **f** Validation of compounds identified in the HTS from **d**, lower left quadrant (blue circle around double hits) and **d** upper left quadrant (red circle around ACh hits) using ACh stimulation in an TRPC6 assay with TRPC6 cells and BTP2 and CPA supplementation (**e**, *p* = 0.29) and in an mAChR assay in HEK wild-type cells with intact Ca^2+^ store release (**f**, *p* = 0.004). In **e**, **f** one dot represents the average half maximal effective concentration (pIC50) of one substance (tested at least eight times). Unpaired, two-sided Student’s *t* test. Data are presented as mean values ±SEM. **p <  0.01; ***p  <  0.001.

In many cases, HTS for new potential drugs acting on intracellular signaling steps faces the problem that hits can be caused by unspecific effects upstream of the target protein. For example, the receptor used for activation of the signaling cascade under investigation might, by itself, be affected by the tested compounds. In consequence, the identification of the target-specific hits requires in-depth evaluation of a high number of compounds in HTS follow-up validation^49^. Thus, an activation step that is not affected by any of the tested compounds would enhance the specificity of positive hits and thereby its efficiency. One important drug target are TRPC6 channels which are physiologically activated by DAG after G_q_ protein-mediated stimulation of PLCβ and which play a major pathophysiological role in many diseases, including glomerulosclerosis and pulmonary hypertension^50^. Drug screening for potential hits inhibiting TRPC6 channels is conventionally performed by either artificial activation using membrane permeable DAG analogs like OAG^50^ or by physiological stimulation of G_q_/PLCβ signaling with acetylcholine (ACh) or ATP to activate endogenous M3 or P2Y receptors, respectively. To prove the advantage of optogenetic stimulation, we developed an all-optical HTS assay for TRPC6 channel inhibitors using hOPN5 instead of ACh for G_q_/PLCβ activation. In HEK cells expressing hOPN5 and TRPC6, UV light-induced Ca^2+^ transients were reduced to approximately 50% of their original amplitude with a delayed onset when removing the IP_3_/ER Ca^2+^ release component by inhibiting the SERCA ATPase and CRAC channels with cyclopiazonic acid (CPA) and bis(trifluoromethyl)pyrazoles2 (BTP2), respectively (Fig. 4a). Blocking these two pathways in hOPN5 expressing HEK cells without TRPC6 expression completely inhibited UV light-induced Ca^2+^ transients (Fig. 4a) thus proving that the remaining non-IP_3_/ER Ca^2+^ signals are specific for Ca^2+^ entry through TRPC6. To further demonstrate that the residual Ca^2+^ flux was selectively mediated via TRPC6, we pharmacologically blocked TRPC6 using SAR 7334^50^. This resulted in a complete block of Ca^2+^ flux (Fig. 4b). Therefore, we performed all further TRPC6 screening experiments in the presence of SERCA and CRAC channel inhibition.

Next, we compared the efficacy of the hOPN5 all-optical screening with the conventional HTS approach of TRPC6 activation by pharmacological ACh stimulation. Interestingly, the dose-response analysis of SAR 7334 showed a slightly higher sensitivity with an IC50 of 22 nM using UV light as stimulation compared to an IC50 of 43 nM when using ACh as agonist (Fig. 4b, c) which is in the range reported previously^50^. We next performed the HTS by testing 218,064 compounds from the Bayer compound library in 1536 multititer plate format to compare the specificity of both assays. We decided to start with UV light for activation of the signaling cascade and then add saturating concentration of ACh (>100 fold over EC50) to render the assay less prone to inhibitors of muscarinic receptors. Overall, 360 substances (0.17%) inhibited TRPC6-dependent Ca^2+^ influx after both hOPN5 and ACh activation. However, 2954 additional substances (1.35%) blocked the Ca^2+^ influx induced only by ACh but not those upon hOPN5 activation (Fig. 4d). Because the potent muscarinic receptor antagonist atropine was among these substances, we speculate that block of muscarinic receptor activation rather than direct action on TRPC6 channels is underlying these false-positive results. In contrast, only three substances were identified to reduce the hOPN5 and not the ACh-induced Ca^2+^ transients. Importantly, these substances inhibited Ca^2+^ transients with rather low efficiency (<50% inhibition) and could not be confirmed in follow-up studies. To validate these HTS results and to differentiate between the presumably false-positive single hits, only affecting ACh-induced Ca^2+^ transients (“ACh hits”), and specific hits diminishing both, ACh and UV light-induced transients (“double hits”), we performed validation assays with representative substances from each group. First, these substances were retested by stimulating HEK cells expressing TRPC6 with ACh in the presence of CPA and BTP-2 (“TRPC6 assay”) confirming their inhibitory activity over a broad range of potencies independent of their mode of action (Fig. 4e). In order to investigate the nature of the false-positive ACh-only hits, we tested the substances on G_q_/PLC-dependent Ca^2+^ release in HEK wild-type cells without TRPC6 expression and without blocking intracellular Ca^2+^ release (“mAChR assay”). In these cells, substances from the ACh-only group blocked Ca^2+^ transients, suggesting that these were unspecific by blocking muscarinergic receptors. Importantly, the double hit substances identified by both HTS screens were not affecting Ca^2+^ transients in these cells that do not express TRPC6 (Fig. 4f) which can be seen by IC50 values well above 10^−5^ M.

Taken together, none of the 218,064 compounds and not even substances classified as frequent hitter or Pan-assay interference compounds (PAINS)^51^ were effectively inhibiting only UV light-induced effects. This suggests that hOPN5 is an ideal activator for G_q_/PLCβ dependent HTS assays (Fig. 4d). Thus, all-optical HTS with hOPN5 is highly specific and enhances the efficiency of HTS screening.

### Positive chronotropic effect of hOPN5 in vitro

**Fig. 5:**
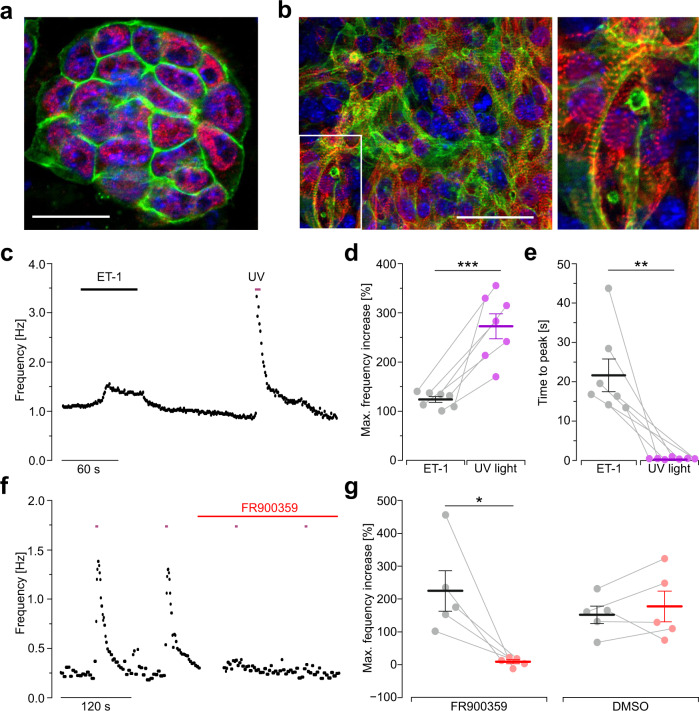
hOPN5 function in mouse embryonic stem cell (ESC) derived cardiomyocytes. **a**, **b** hOPN5 ESC identified by Oct3/4 staining (**a**, red) and an embryoid body (EB, **b**) containing α-actinin positive cardiomyocytes (red) show membrane-bound eYFP signals (green) indicating hOPN5 expression (nuclear staining with DAPI in blue, bar = 25 µm **a**, 50 µm **b**). **c**–**e** Frequency analysis of spontaneous beating of hOPN5 EBs. Representative frequency time course (**c**) during pharmacological stimulation with 100 nM Endothelin 1 (ET-1, black) and UV light (385 nm, 300 µW/mm^2^, 5 s, violet) and statistical analysis of the maximum frequency increase (**d**, *p* < 0.001) and time to peak (**e**, *p* = 0.002). Each dot represents the result from one individual EB. **f** Representative time course of the beating frequency with UV light pulses (300 µW/mm^2^, 5 s, violet) before and after application of FR900359 (1 µM, red bar, break represents 10 min incubation time). **g** Statistical analysis of maximum frequency increase before and after FR900359 or DMSO application. Statistical analysis was performed with two-way paired Student’s *t* test (p(DMSO) = 0.492). Data are presented as mean values ±SEM. *p <  0.05, **p <  0.01, ***p  <  0.001.

To explore the potential of hOPN5 for cardiac optogenetic applications, we generated transgenic mouse embryonic stem cells expressing hOPN5 in fusion with eYFP under the control of the chicken-β-actin promoter since this promoter is highly active in stem cells and differentiated cardiomyocytes^21,52^. In stem cells (Fig. 5a) as well as in differentiated cardiomyocytes within embryoid bodies (Fig. 5b) expression of hOPN5/eYFP was targeted to the cell membrane. Because the pacemaking machinery in these cardiomyocytes is controlled by the G_q_-IP_3_ signaling cascade^53^, we recorded spontaneous beating of cardiomyocyte containing embryoid bodies and compared UV light stimulation to pharmacological stimulation with Endothelin 1 (ET1), an agonist of the G_q_ coupled ET1 receptors which are expressed in these cardiomyocytes. Both treatments led to an increase in the spontaneous beating rate (Fig. 5c) but stimulation with UV light induced a stronger and much faster effect (Fig. 5d, e). This proves the higher temporal precision of optogenetic stimulation since light stimulation is not depending on perfusion and diffusion kinetics of agonists. To prove that the light-induced beating rate increase is selectively caused by G_q_ signaling, we applied the specific blocker FR900359, which fully abolished the light effect (Fig. 5f, g). Importantly, after blocking G_q_ proteins, we also could not observe a light-induced decrease in the spontaneous beating rate, ruling out possible co-activation of the G_i_ pathway. In conclusion, hOPN5 activates G_q_ proteins in cardiomyocytes differentiated from embryonic stem cells and we could not detect any promiscuity towards G_i_ signaling.

### hOPN5 specifically activates G_q_ proteins in adult intact hearts

**Fig. 6:**
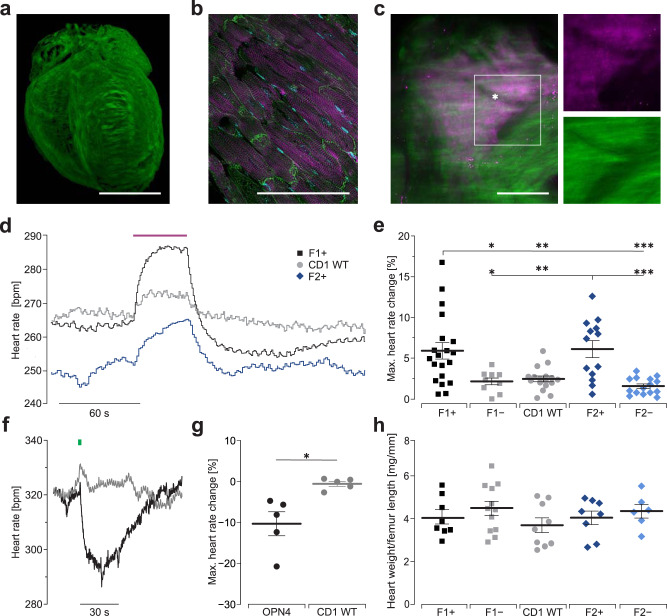
Modulation of heart rate in hOPN5/eYFP and OPN4/eGFP transgenic hearts. **a**–**c** Distribution of hOPN5/eYFP (green) expression in the whole heart (**a**), in a ventricular section with α-actinin (purple) positive cardiomyocytes (**b**, DAPI in cyan) and in the sinus node identified by HCN4 staining (**c**, purple). Note the sinus nodal artery as classical landmark (**c**, star). Bars = 3 mm **a**, 100 µm **b**, 300 µm **c**. **d** Representative heart rate traces from hearts of hOPN5/eYFP founder line #1 and #2 with hOPN5 expression (F1+, F2+), and wild-type control mice (CD1 WT) upon illumination of the dorsal right atrium with UV light (385 nm, 1 mW/mm^2^, 10 s). **e** Statistical analysis of the light induced maximum heart rate increase in hOPN5/eYFP hearts (F1+, F2+) and wild-type hearts (CD1 WT) as well as hOPN5 negative littermates (F1−, F2−) from both founder lines. Each dot represents the average from one heart and statistical analysis was performed with a one-way non-parametric ANOVA test with Tukey’s multiple comparison post-test (p(F1+ vs. F2+): 0.99; p(F1− vs. CD1 WT): 0.99; p(F1− vs. F2−): 0.99; p(CD1 WT vsF2−): 0.91). **f** Representative time course of changes in beating rate in an OPN4/eGFP (black) and a CD1 WT heart (gray) upon blue light (460 nm, 1 s, 1.4 mW/mm^2^) application to the same region as in **d**. **g** Statistical analysis (*n* = 5) of maximal change in heart rate using two-sided unpaired Student’s *t* test (*p* = 0.01). **h** Analysis of the heart weight to femur length in 2-months-old hOPN5/eYFP mice with a ordinary one-way ANOVA test with Tukey’s multiple comparison post-test (p(F1+ vs. F1−): 0.88; p(F1+ vs. CD1 WT): 0.93; p(F1+ vs. F2+): 0.99); p(F1+ vs. F2−): 0.98; p(F1− vs. CD1 WT): 0.37; p(F1− vs. F2+):0.86; p(F1− vs. F2−): 0.99; p(CD1 WT vs. F2+): 0.94; p(CD1 WT vs. F2−): 0.71; p(F2+ vs. F2−): 0.97). Each dot represents the result from one heart. Data are presented as mean values ±SEM. *p <  0.05, **p <  0.01, ***p  <  0.001.

In contrast to embryonic stem cell-derived cardiomyocytes, the adult heart has very pronounced physiological G_i_ signaling effects important for parasympathetic heart rate deceleration at rest. Thus, we consider the adult heart as an ideal cellular system to detect promiscuous G_i_ signaling by hOPN5. We generated a transgenic mouse model expressing hOPN5 in fusion with eYFP in the heart using the chicken-β-actin promoter. Indeed, in two separate founder lines, we found strong membrane-restricted expression of hOPN5/eYFP in cardiomyocytes equally distributed throughout the whole heart (Fig. 6a, b and Supplementary Video 2). No eYFP expression was found in vessels. Analysis of isolated ventricular cardiomyocytes revealed that 60 ± 11% (*n* = 5) and 68 ± 7% (*n* = 5) of the cardiomyocytes expressed hOPN5/eYFP in founder line F1 and F2, respectively (*p* = 0.16, unpaired Student’s *t* test). Importantly, we found strong expression within the sinus node identified by HCN4 staining (Fig. 6c and Supplementary Video 3) which allows to distinguish unambiguously, between activation of G_i_ proteins leading to a decrease in the spontaneous beating rate^54^ and activation of G_q_ proteins increasing the spontaneous beating rate via the Ca^2+^ clock machinery^55,56^. For this purpose, hearts were explanted, retrogradely perfused through the aorta in Langendorff configuration and illumination was focused on the posterior part of the right atrium for specific stimulation of the sinus node region. In transgenic hearts from both founder lines, UV light (385 nm, 10 s, 1 mW/mm^2^) induced an increase in the spontaneous beating rate which was significantly higher compared to effects in hearts from non-transgenic siblings as well as in CD1 wild-type mice (Fig. 6d, e). It is of note that a mild increase in the beating rate by UV light was also observed in wild-type control hearts. The effect was more pronounced at increasing light intensities and is most likely due to heating effects.

To compare the effects by hOPN5 to those of the G_i_/G_q_ promiscuous light-sensitive receptor Melanopsin (OPN4), we generated another transgenic mouse model expressing OPN4 and GFP under the control of the identical chicken-β-actin promoter. In hearts from those mice, illumination (470 nm, 1 s, 1.4 mW/mm^2^) of the same region as for the hOPN5/eYFP mice reduced the spontaneous beating rate by 10.3 ± 2.9% (*n* = 5) (Fig. 6f, g), rather than accelerating it. This direct comparison to OPN4 in the exactly same cellular context proves the high specificity of hOPN5 for G_q_ proteins.

Side effects by hOPN5/eYFP overexpression were excluded with basal electrogram recordings which did not show significant differences in the beating rate, PR interval indicating atrial and AV nodal conduction, QRS interval indicating the electrical activation of the ventricles as well as the QT interval, an average of ventricular action potential duration (Supplementary Fig. 2). Because chronic G_q_ activation was shown to induce pathological cardiac hypertrophy^57^, we measured heart weight to femur length in two months old mice and did not detect any differences between hOPN5/eYFP mice, sibling controls, and wild-type mice (Fig. 6h). Taken together, hOPN5 can be used to investigate G_q_ signaling in the adult heart without noticeable overexpression side effects.

**Fig. 7:**
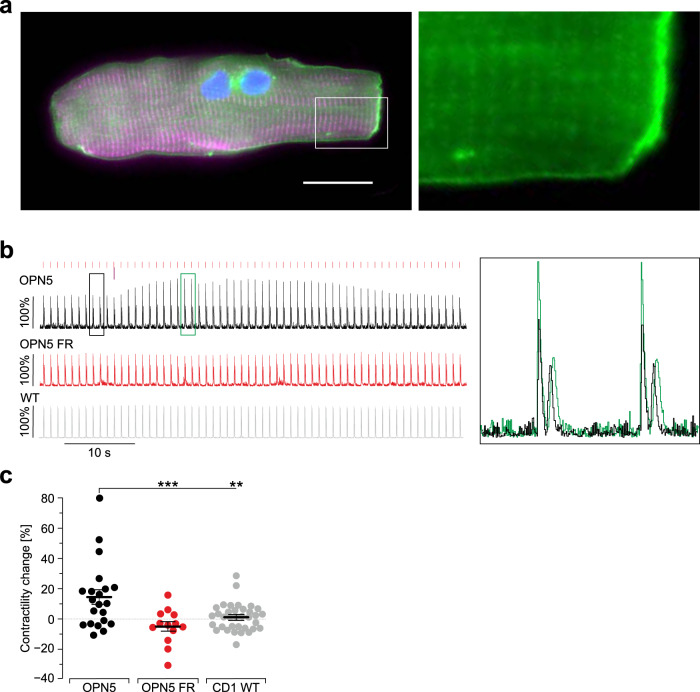
hOPN5 increases contractility in adult ventricular cardiomyocytes. **a** Adult α-actinin (purple) positive ventricular cardiomyocyte from an hOPN5/eYFP transgenic heart with eYFP signals (green) in the membrane and t-tubule invaginations (DAPI in blue, bar = 20 µm). **b** Representative trace showing the contractility analysis (average pixel displacement per frame) of ventricular cardiomyocytes expressing hOPN5 before (black) and after application of FR900359 (1 µM, red) as well as of a control cardiomyocyte from a CD1 wild-type mouse (gray) during electrical pacing (1 Hz, red lines) with one UV light pulse (385 nm, 1 mW/mm^2^, 100 ms, violet arrow). Insert highlights the stronger contraction after (green) compared to before (black) illumination. **c** Statistical analysis of contractility change (each dot represents the result from one cardiomyocyte, isolated from 4 mice for hOPN5 and 3 for CD1 wild-type control mice) was performed with a non-parametric ANOVA test with Tukey’s multiple comparison post-test (p(WT vs hOPN5 FR) = 0.4). Data are presented as mean values ±SEM. **p <  0.01, ***p  <  0.001.

To further demonstrate G_q_ effects in the adult ventricular cardiomyocytes, we investigated effects on contractility. For this purpose, we isolated ventricular cardiomyocytes from adult mouse hearts and analyzed their isotonic contraction with a custom-made software allowing online analysis of contractility using optical flow analysis. hOPN5/eYFP expressing cardiomyocytes were identified by their membrane-bound eYFP fluorescence signal (Fig. 7a) and contractions were analyzed during continuous electrical pacing at 1 Hz. UV light flashes induced a significant increase in contraction amplitude (Fig. 7b). This effect was fully abolished after incubation with FR900359 and was not observed in control cardiomyocytes isolated from CD1 wild-type mice (Fig. 7b, c).

Altogether, the data from the transgenic mouse model proves the specificity of hOPN5 for G_q_ signaling and its value for optogenetic application in cardiomyocytes and the heart.

### hOPN5 in smooth muscle cells

**Fig. 8:**
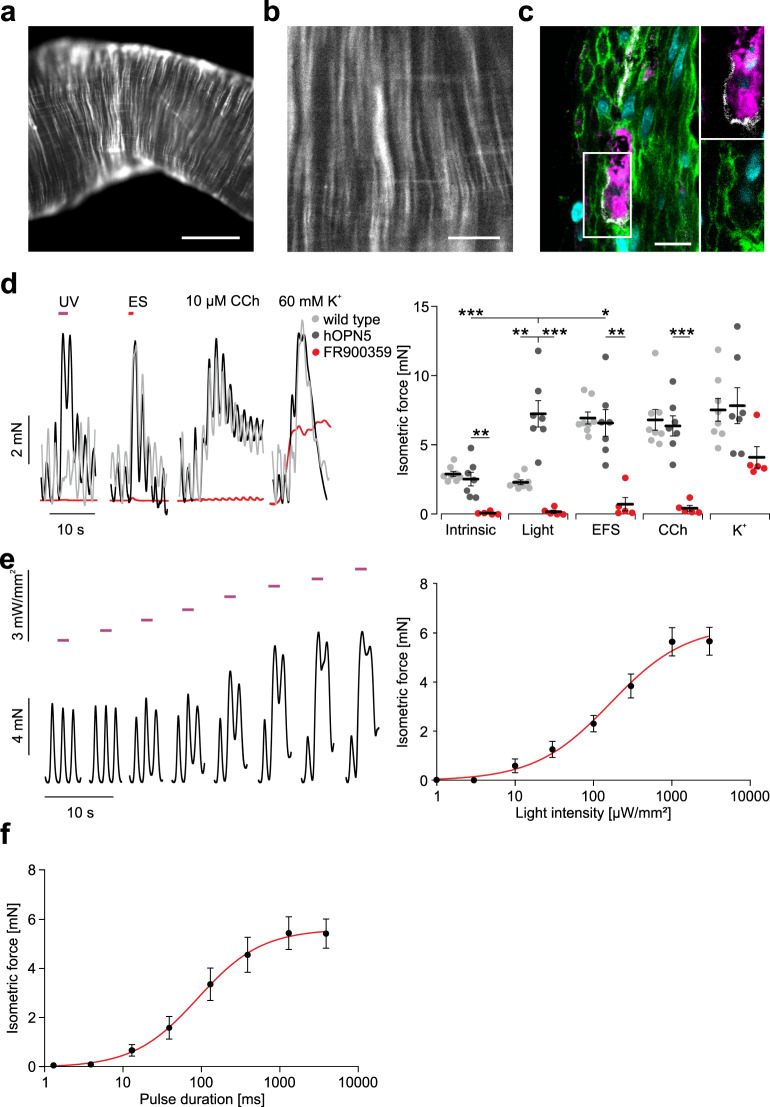
UV light induced contractions in small intestine of hOPN5 mice. **a**, **b** eYFP-fluorescence (white) in longitudinal as well as circular orientation in the small intestine of an hOPN5 mouse. **c** hOPN5/eYFP signals (green) in the tunica muscularis of the small intestine cannot be detected in c-kit positive Interstitial cells of Cajal (white) and β-III-Tubulin positive neurons (purple). Nuclear staining with DAPI (cyan). Bar = 2 mm in **a**, 400 µm in **b**, and 10 µm in **c**. **d** Representative traces (left) and aggregated data (right) of isometric force measurements of explanted small intestine from CD1 wild-type controls (*N* = 3, *n* = 8; gray) and hOPN5 mice (*N* = 3, *n* = 7) before (black) and after application of FR900359 (red, 1 µM, *N* = 3, *n* = 5). Typical intrinsic contraction patterns can be observed and additional contractions were induced by UV light (violet bar, 2 s, 3 mW/mm^2^), EFS (red bar, 1 s, 100 Hz, 2 ms pulses, 40 V), 10 µM CCh and 60 mM K.^+^ Statistical analysis was performed with two-way repeated measures ANOVA with Sidak’s multiple comparison test between CD1 WT vs. hOPN5, CD1 WT vs. hOPN5 FR and hOPN5 vs. hOPN5 FR (p(UV light): 0.0023, 0.0009, and 0.01, respectively; p(intrinsic): 0.62, 0.0009, and 0.04; p(EFS): 0.78, 0.005, and 0.03; p(CCh): 0.61, 0.01, and 0.007; p(K^+^): 0.94, 0.15, and 0.29) and Tukey’s multiple comparison test for light effects in comparison to other stimuli in hOPN5 samples (p(vs. intrinsic): 0.0006; p(vs. EFS): 0.048; p(vs. CCh): 0.09; p(vs. K^+^): 0.93). **e** Representative force traces (left) with 2 s long UV light pulses (violet bars) with increasing light intensity (from left: 1, 3, 10, 30, 100, 300, 1000, and 3000 µW/mm^2^, light intensities displayed on a logarithmic scale) and aggregated data (right) of the light intensity to force relationship with a Hill fit (red). **f** Analysis of pulse duration to force relationship for light pulses of constant intensity (3 mW/mm^2^) with a Hill fit (red). **d**, **e**: *N* = 2 mice; *n* = 5 stripes. Data are presented as mean values ±SEM. *p <  0.05, **p <  0.01, ***p  <  0.001.

To further show the potential of hOPN5 for optogenetic use, we investigated hOPN5-dependent G_q_ activation in smooth muscle cells within three organs. The proximal parts of the small intestine showed bright eYFP signals in the longitudinal and circular smooth muscle layer (Fig. 8a, b) which was restricted to smooth muscle cells (Fig. 8c). Because G_q_ is the major signaling cascade for smooth muscle contraction, we performed isometric force measurements. Only in hOPN5/eYFP expressing intestine, illumination with UV light resulted in transient increase of force which was higher than electrical field stimulation and similar to pharmacological stimulation with CCh or depolarization by high K^+^ concentrations. Since also G_i_ activation can increase force in smooth muscle cells by lowering cAMP concentrations, we applied the G_q_ specific blocker FR900359, which prevented UV light-induced force generation as well as the G_q_ mediated electrical field and CCh stimulaion (Fig. 8d). Repetitive illumination with either increasing light intensities (Fig. 8e) or increasing pulse duration (Fig. 8f) demonstrates the fine-grain control over force generation by optogenetic stimulation. Detailed analysis revealed a half maximal effective light intensity (eLi50) of 140 ± 24 µW/mm^2^ (*N* = 2, *n* = 5) for 2 s (280 µW*s/mm^2^) long light pulses and half maximal effective pulse duration of 87 ± 22 ms (*N* = 2, *n* = 5) at 3 mW/mm^2^ (261 µW*s/mm^2^).

**Fig. 9:**
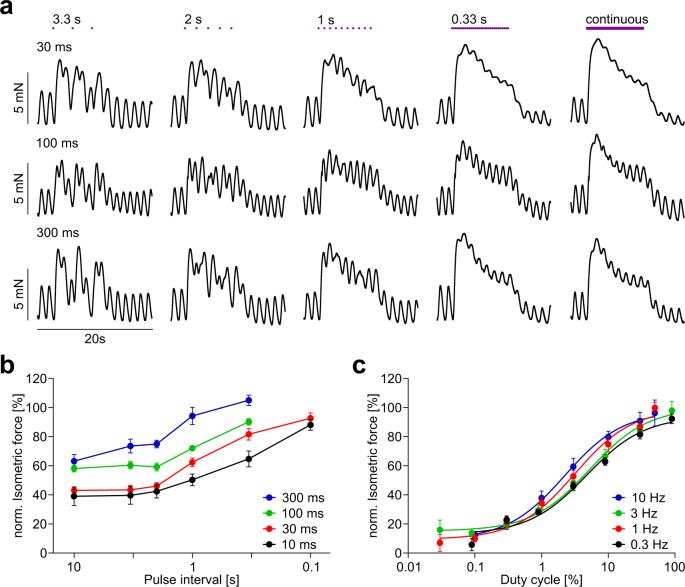
Efficacy of pulsed illumination in the small intestine. **a** Representative traces of isometric force using different illumination patterns (violet dots, 1 mW/mm^2^) with decreasing pulse intervals (3.3 s, 2 s, 1 s, 0.33 s and continuous light) at different pulse lengths (30 ms, 100 ms, 300 ms). **b** Aggregated data of the dependence of the normalized maximal isometric force amplitude on the pulse interval at various pulse durations (*N* = 2, *n* = 8). **c** Analysis of duty cycle to isometric force relationship when using 0.3 (black, *N* = 2, *n* = 8), 1 (red, *N* = 3, *n* = 12), 3 (green, *N* = 3, *n* = 12), and 10 Hz (blue, *N* = 3, *n* = 12) repetition rates with changing pulse durations (1 mW/mm², 10 s long illumination pattern). Peak isometric force was normalized to the ones from continuous illumination, shown is the mean and the respective Hill fit. Data are presented as mean values ±SEM.

To explore superposition and refractoriness in more detail, we applied pulses of various durations and repetition rates (Fig. 9a). At light pulse intervals smaller than 2 s, contractions were superimposed and summed up with maximal force generation under continuous illumination (Fig. 9a, b). As a consequence, overall force generation depends mainly on product of pulse duration and repetition rate (duty cycle) of the illumination (Fig. 9c) with very similar half maximal values for all tested repetition rates (5.8 ± 1.1% for 0.3 Hz (*N* = 2, *n* = 8), 4.7 ± 1.6% for 1 Hz (*N* = 3, *n* = 12), 10 ± 3.8% for 3 Hz (*N* = 3, *n* = 12), and 4.2 ± 1.3% for 10 Hz (*N* = 3, *n* = 11, non-repeated measurements one-way ANOVA, *p* = 0.28). Approximately 30% duty cycle was sufficient to induce near maximal forces (Fig. 9c).

**Fig. 10:**
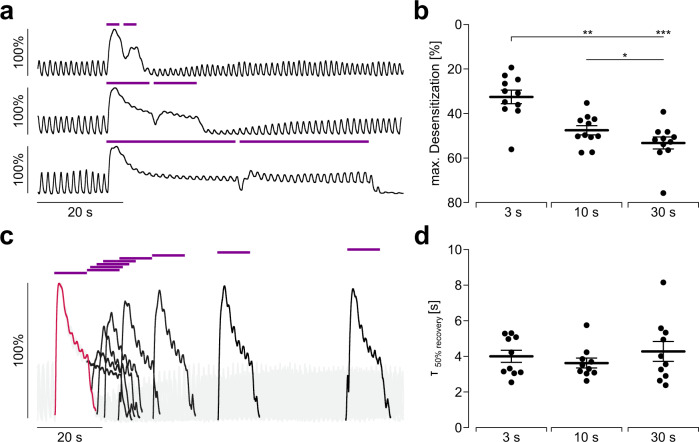
Desensitization and recovery from desensitization of light-induced force in the small intestine. **a**, **b** Representative traces (**a**) and aggregated data (**b**) of isometric force measurements showing the maximum desensitization (relative decrease) of light-induced isometric force in dependence of the applied pulse duration (violet bars, 1 mW/mm^2^, from top to bottom: 3 s, 10 s, 30 s). Statistical analysis was performed with repeated measurements one-way ANOVA with Tukey’s multiple comparison test (*N* = 4, *n* = 11; p(3 vs. 10 s): 0.0015; p(3 vs. 30 s): 0.0005; p(10 vs. 30 s): 0.04). **c** Overlay of representative traces of a paired pulse protocol with 10 s long UV light pulses (violet bars, 1 mW/mm^2^) at increasing intervals (0.1 s, 1 s, 3 s, 5 s, 10 s, 20 s, 40 s, 80 s). Force induced by the first pulse is highlighted in red and the force generated by the second light pulses is shown in black. **d** Aggregated data showing the half maximal recovery time at different pulse durations. Statistical analysis was performed with ordinary one-way ANOVA (*N* = 4, *n* = 10, *p* = 0.56). Data are presented as mean values ±SEM.

Next, we tested the effect of pulse duration using prolonged, continuous illumination. The extent of desensitization of hOPN5 effects increased with prolonged pulse durations (Fig. 10a, b) whereas the time constant of recovery from desensitization was similar throughout all tested pulse durations (Fig. 10c, d). Interestingly, the recovery of light-induced force generation was shorter than the recovery of the spontaneous contraction patterns (Fig. 10c).

In addition to the small intestine, hOPN5/eYFP was also well expressed in the uterus and the bladder, which both showed UV light-induced force generation (Supplementary Fig. 3). Importantly, spontaneous intrinsic contraction patterns as well as responses to electrical field stimulation, pharmacological stimulation with CCh and depolarization by high K^+^ concentrations did not differ between the three organs from hOPN5/eYFP mice and CD1 wild-type mice excluding side effects by hOPN5/eYFP overexpression (Fig. 8d and Supplementary Fig. 3). Taken together, we can demonstrate the use of hOPN5 as optogenetic tool to control smooth muscle contractility.

## Discussion

Herein, we demonstrate the use of hOPN5 for optogenetic applications. We report a detailed analysis of hOPN5 function in several cell types as well as in a transgenic animal model. In contrast to previous reports^34,35,39,44^, we could not find any indication of G_i_ protein activation and prove with several approaches in cell models and intact organs, specific blockers as well as knock out models the specific and selective activation of G_q_ signaling. Importantly, we used the GIRK channel assay and the influence on the spontaneous beating rate in the intact adult heart since both are very sensitive for G_i_ signaling and activation of G_i_ or G_q_ proteins lead to opposite effects. Thus, both assays allow to detect even minimal promiscuity or prove the specificity towards one or the other G protein within the physiological environment without any pharmacological or genetic alterations. Our functional experiments are superior to previous simplified radioactive ligand binding assays in a non-physiological context^34,35,39^ where the high abundance of G proteins can overcome the functionally essential differences in G protein affinity. Furthermore, such in vitro reconstitution assays using purified proteins are lacking the normal cellular environment and thus potential phosphorylation or glycosylation^34^ and the influence from surrounding microdomains. In this study, we could not reproduce the previously reported inhibition of adenylylcyclases and decreases in cAMP production in HEK cells by hOPN5 activation^39,44^. The former observations can either be explained by differences in expression techniques or by G_q_ protein dependent elevation of Ca^2+^ levels which is known to inhibit the adenylylcyclases 5 and 6^58^, the main isoforms in HEK cells^59^. Although activation of G_q_ proteins has been suggested for hOPN5 and chicken OPN5^38,44^, it has not been proven yet by specific blockers for the G_q_ family proteins G_q_, G_11_, and G_14,_ such as FR900359. In addition, we found that light induced Ca^2+^ transients were abolished in G_q/11_ KO cells proving that hOPN5 is acting through at least one of these two subunits. The herein proven specificity of hOPN5 for G_q_ signaling is not only important for optogenetic applications but will be of importance for the interpretation of its physiological role in the various hOPN5 expressing tissues^36–38,41–43,60^. HEK cells overexpressing hOPN5 without a fluorescence reporter at the C-terminus showed very similar Ca^2+^ responses and did not alter activation behavior. However, we cannot exclude that the specificity towards G_q_ signaling is a result of the C-terminal eYFP tag and that there might be still some G_i_ protein activation which we are not able to detect. It is also important to note that the specificity towards G_q_ proteins has to be proven for other cell types than those investigated in this study. Taken together, hOPN5 is a very specific activator of G_q_ signaling and has a high potential for optogenetic application.

The selective activation of G_q_ signaling by UV light can be an advantage for experiments in cellular systems since it allows the simultaneous optical recording of intracellular Ca^2+^ dynamics and other second messengers as shown with Fluo-8, X-Rhod-1-AM, and GCaMP6. More generally, a wide variety of functional indicators, such as membrane voltage sensors or sensors for catecholamines are compatible with hOPN5 using green to red excitation light. We, for our part, could not observe any effects of the low-intensity imaging light on hOPN5 activation. However, we cannot completely exclude that imaging light had an impact on the duration of the activated state of hOPN5 and could accelerate its inactivation. The light sensitivity of hOPN5 is in the optimal range since it is not activated by normal laboratory light and the required light intensities and pulse durations are below toxic light energies and did not negatively affect cell or organ function. Using paired pulse protocols, we can observe desensitization of G_q_ stimulation which is depending on the duration of hOPN5 activation. However, recovery from desensitization is not dependent on the pulse duration and 50% recovery is already seen after ~4 s. Interestingly, we saw that in the small intestine the recovery of spontaneous phasic activity took longer than the one of UV light-induced contractions. Thus, kinetics of optogenetically induced force stimulation are faster than that of the biological system and are thus well suited to study kinetic properties of force generation. This may offer the possibility to investigate the mechanisms underlying the temporal superposition of contractions that have only scarcely been studied in smooth muscle tissues so far. Moreover, existing studies were largely dependent on indirect activation of smooth muscle cells via stimulation of intramural neuronal varicosities^61–63^.

Despite the fact that we were able to prove the bi-stability of hOPN5 using a fast paired-pulse protocol with different wavelengths, it seems that the intrinsic relaxation from the active all-trans retinal form back to the deactivated state was very fast: Even without applying green/yellow light, all light effects stopped immediately after the end of UV illumination. Therefore, to use hOPN5 as a functional bi-stable optogenetic receptor that can be switched on by UV and off by yellow light, hOPN5 must be mutated to slow spontaneous relaxation. Common strategies using single amino acid exchanges to enhance the bi-stability of photoreceptors have been successfully applied for different optogenetic proteins^64,65^. Despite the low tissue penetrance of UV light, the high light sensitivity of hOPN5 enabled its use in the heart as well as the small intestine, the uterus and the bladder tissue. Still for specific optogenetic applications, hOPN5 or optoXR chimera^5,33^ including specific hOPN5 sequences can be created to shift the activation wavelength towards the red spectrum.

Importantly, having an optogenetic receptor at hand which is commonly expressed in humans provides a unique opportunity for translational approaches because ectopic expression most likely does not trigger immune responses^66,67^. Increasing activity of G_q_ signaling with hOPN5 expression and illumination might help to increase the efficiency of optogenetic strategies^32^, specifically those targeting smooth muscle contractility^68,69^. We could not detect any side effects by sole overexpression of hOPN5 in cells and organs which excludes dark activity of the receptor or alterations of the native G_q_ signaling cascade.

We further demonstrate an important biotechnological application of hOPN5 by establishing all-optical HTS for potential blockers of target proteins activated by G_q_ signaling, in this case TRPC6 channels. In general, all-optical HTS provides the advantages of contact-free and repetitive activation of G_q_ signaling over prolonged periods of time. This is especially important for HTS settings which are conventionally limited regarding efficient exchange of compound or agonist containing solutions during HTS operation. Reducing liquid handling steps required to activate signaling in a screening campaign will increase throughput and reliability of the robotic testing, decrease statistical noise and thus very likely reduce the numbers of false positive or false negative results. In our case, hOPN5 itself turned out to be almost untargetable by the test compounds; not even by compounds classified as “frequent hitters” and “Pan-assay interference compounds”^51^. As a consequence, the all-optical approach proved to be much more specific and slightly more sensitive. Thus, the herein demonstrated all-optical HTS demonstrates the general power to find new drugs altering G_q_ signaling, one of the major signaling cascades in almost every cell type and organ of the human body.

Taken together, we provide clear evidence for the high specificity of hOPN5 towards G_q_ proteins raising a tantalizing outlook for optogenetic approaches in cells, drug screening, organs and even in vivo applications.

## Methods

### Ethical statement

All animal work conformed to the European Guideline for animal experiments 2010/63/EU and the mouse generation was approved by the Niedersächsische Landesamt für Verbraucherschutz und Lebensmittelsicherheit (approval number 33.9-42502-04-16/2352) and by the Landesamt für Natur, Umwelt und Verbraucherschutz Nordrhein-Westfalen (approval number AZ.84-02.04.2012A146). Mice were kept in 12/12 h dark/light cycles with food and water ad libido at room temperature and standard humidity.

### Generation of hOPN5 constructs and transgenic HEK293 cell lines

The codon optimized gene sequence of human Neuropsin OPN5 (hOPN5) was ordered from GeneArt Gene synthesis (Thermo Fisher Scientific, U.S.) and inserted after digestion with SacI and NotI into plasmids previously reported, for expression of hOPN5 either in fusion at the C-terminus to eYFP (hOPN5/eYFP) or using an IRES sequence for non-targeted cytosolic GFP (hOPN5 IRES GFP) under the control of the chicken-β-actin promoter. Expression of the muscarinergic M2 receptor in fusion with CFP was performed using a plasmid reported before^70^. The plasmid also comprises a eukaryotic Neomycin/G418 resistance cassette.

HEK cells (AD293, ATCC, U.S.) and G_q/11_^45^ and G_i_ KO cells^46^ were cultured in Dulbecco’s Modified Eagle’s Medium (DMEM, Thermo Fisher Scientific) supplemented with 10% FCS (PAN-Biotech, Germany), 0.1 mmol/L nonessential amino acids (Thermo Fisher Scientific), 100 U/mL penicillin, 100 mg/mL streptomycin (Thermo Fisher Scientific) and 0.1 mmol/L β-mercaptoethanol (Th. Geyer, Germany) in T25 flasks (Sarstedt, Germany) and passaged at ~1:20 ratio using Trypsin (PAN-Biotech) when a density of 70–90% was reached. Cells were transfected using Fugene 6 transfection reagent (Promega, US) according to the manufacturer’s protocol and selected by adding 900 µg/mL G418 (Thermo Fischer Scientific) with medium exchange every second day. For maintenance of transgenic HEK cell lines, G418 concentration was reduced to 600 µg/mL. To obtain single cell clones, we plated cells at a density of 0.3 cells per well into a 96 well plate (Sarstedt) and checked the fluorescence signals before further passaging. For culture of HEK cells stably expressing Girk1/2^71^, the T25 flasks were coated with 0.1 % gelatine and these cells were kept under 3 µg/mL Puromycin (Th. Geyer) and 5 µg/mL Blasticidin (InvivoGen, U.S.) for maintenance. In these cells, hOPN5 expression and selection were performed as described above.

### IP_1_ assay

IP_1_ concentrations were measured with the homogeneous time-resolved fluorescence competitive immunoassay (IP-One - G_q_ HTRF kit, Cisbio, U.S.) according to the manufacturer’s instructions. Briefly, 10,000 monoclonal hOPN5/eYFP or wild-type HEK cells were plated on a half area 96 well plate (1074306, CELLSTAR®, Greiner Bio-One, Germany) coated with 10 µg/mL fibronectin (Sigma-Aldrich) and cultured overnight. Prior to the experiment, we added 2 µM all-trans-retinal (ATR, R2500, Sigma-Aldrich) and the experiments were performed in complete darkness at 37 °C. Illumination with UV light (50 µW/mm^2^, 1 s, every 5 min for 30 min) was performed through an inverted fluorescence microscope Olympus IX83 equipped with a RTC MT20 lamp, a 4x UPLSAPO objective (0.16 NA) and a DAPI Filter (387/11 Excitation and AT415 dichroic mirror, AHF Analysetechnik, Germany) controlled by the CellSens® Dimensions software (Olympus, Japan). Cells were treated with 300 µM of the muscarinic agonist Carbamoylcholine chloride (CCh, Sigma-Aldrich) as a positive control and G_q_ signalling was blocked with the pan G_q_ blocker 1 µM FR900359^45^ added 10 min before the experiment. IP_1_ levels were measured in the cell lysate after 30 min in a 384 well Polystyrene microplate (781074, Greiner Bio-One) using the CLARIOstar® microplate plate reader (BMG Labtech, Germany). The IP_1_ concentration was calculated by correlation of the 665/620 nm ratio to a prior determined IP_1_ standard curve.

### Ca^2+^-imaging

HEK cells, wild-type and polyclonal hOPN5/eYFP, were plated on 22 × 22 mm glass plates coated with 10 µg/mL fibronectin. Cells were incubated with 2 µM ATR and loaded with 1.5 µM X-Rhod-1-AM with 1x PowerLoad™ Concentrate (Thermo Fisher Scientific) for 20 min at room temperature with a subsequent de-esterification step for 20 min at 37 °C in Tyrode solution containing in mM: 1.8 CaCl_2_, 140 NaCl, 5.4 KCl, 2 MgCl_2_, 10 Glucose, 10 HEPES; pH adjusted to 7.4 using NaOH (all substances from Sigma-Aldrich). Experiments were performed at 35 °C and constant perfusion of the cells on an inverted IX73 microscope (Olympus) equipped with a 20x objective (LUCPLFLN20XPH/0,45). Illumination was performed with a LEDHub (Omicron, Germany) equipped with a UV-LED (385 nm) with a 10% neutral density filter and a green light LED (500–600 nm) with a 549/15 nm bandpass filter (AHF Analysetechnik). The light of the LEDHub was coupled into the microscope via a 2 mm light guide (NA 0.5) and a dual port microscope coupler (Cairn Research, UK) and directed onto the objective with a HC BS 561 nm beamsplitter. Emission of X-Rhod-1-AM was collected through a 709/167 nm bandpass filter (AHF Analysetechnik), imaged using a PCO edge 4.2 camera (PCO AG, Germany) and the µManager open source microscopy software (Version 2.0-gamma). Timing of LEDs and the camera was synchronized with a PowerLab 8/35 system and the LabChart 8.1.16 software (AD Instruments, Australia). Pictures were analyzed with the ImageJ 1.52p open source software and the amplitude of the Ca^2+^ transients were defined by the highest signal within the 17 s after the light pulse or the peak amplitude induced by 2 mM CCh and 1.5 mM Adenosine 5‘-triphosphate magnesium salt (ATP, Sigma-Aldrich) applied through the perfusion system within the average F/F0 traces of one ROI comprising at least 20 cells. Desensitization and the recovery from desensitization of light-induced Ca^2+^ transients were investigated by paired pulses with 1, 3, and 10 s pulse duration (1 mW/mm^2^) with interpulse interval increasing from 180, 240, 300 to 400 s. Pictures were analysed with both ImageJ 1.52p and CellProfiler 4.2.1 (open-source software, US) and maximum amplitude of the second Ca^2+^ transients normalized to the first one.

Wavelength sensitivity for activation and inactivation was investigated with an Axiovert 200 microscope (Zeiss, Germany), a 20x objective (20x Fluar, NA: 0.75, Zeiss) and monochromic light pulses (30 ms, 15 nm bandwidth) generated by a computer-controlled monochromator (Polychrome 5, TILL Photonics, Germany) was merged with X-Rhod-1-AM excitation light from a 500–600 nm LED (LEDHub, 572/28 nm excitation filter) using a dual port condenser (TILL Photonics) and a HC BS 561 nm DCLP dichroic filter. Light was directed to the objective with a T580 LPXR (F48-580) dichroic mirror and emission light passed a 594 nm long pass filter (AHF Analysetechnik) and was recorded with Andor Luca EM S camera (Oxford Instruments, UK) and the Live acquisition software (FEI Imaging, Germany). Bistability/light dependent inactivation of hOPN5 was investigated by a double light pulse protocol consisting of one supramaximal UV light pulse (390 nm, 1.2 mW/mm^2^, 30 ms) directly (~4 ms delay) followed by a second 300 ms long light pulse of varying wavelengths. Inhibition of hOPN5 by the second light pulse was quantified as the Ca^2+^ transient amplitude induced by the respective light pulses normalized to the Ca^2+^ transient after sole UV light illumination and displaced as the percentage of inhibition.

### Measurement of cAMP kinetics

hOPN5/eYFP and M2/CFP HEK293 cells were plated at 20,000–40,000 cells per well in 96 well plates (Greiner Bio-One 655094) coated with 10 µg/mL fibronectin (Sigma-Aldrich). 24 h later the GloSensor™-22F plasmid (Promega) was transiently transfected with FuGENE® HD (12.5 ng DNA/μl) in Opti-MEM® I (Invitrogen) accordingly to the manufacturer´s instruction. 20–24 h later 200 µl equilibration medium was applied to each well containing 88% CO_2_-independent medium, 10% fetal bovine serum 2% GloSensor™ cAMP reagent stock solution (Promega) and 10 µM all-trans-retinal (Santa Cruz Biotechnology). 2 h later luminescence measurements (1 s integration, 90 s cycle time) were performed at room temperature in an Infinite® 200 plate reader (Tecan) modified with a light guide in the injector port for application of LED light (385 nm, LEDMOD, Omicron Laserage, Germany) as reported before^72^. After baseline measurements, Forskolin (10 µM, Sigma Aldrich) was applied and the luminescence increase was monitored. Illumination of hOPN5 cells (500 ms, 110 µW/mm^2^) or application of Carbachol (CCh, 100 µM, Sigma Aldrich) to muscarinergic 2 (M2) receptor cells were performed in the decay phase after reaching the cAMP maximum. Effect on cAMP leves were analyzed with a linear decay fit.

### Patch clamp

HEK GIRK1/2 expressing hOPN5/eYFP or control cells were plated at low density on fibronectin-coated (10 µg/mL) coverslips and cultured in above described conditions. 2 µM ATR was added to the medium overnight and patch clamp experiments were performed within two days using an EPC10 amplifier (Heka, Germany) in the whole cell configuration at room temperature with 10 kHz sampling rate on an Axiovert 200 microscope, 20x objective (Zeiss, see above for optical configuration). External solution comprised (in mM) 140 NaCl, 20 KCl, 1 CaCl_2_, 1 MgCl_2_, 10 HEPES, 10 Glucose; pH adjusted to 7.4 using NaOH, internal solution 20 NaCl, 130 KCl, 1 MgCl_2_ 10 HEPES, 5 EGTA, 2 MgATP, 0.3 Na_2_GTP; pH adjusted to 7.2 using KOH (all Sigma-Aldrich). Cells were kept at −40 mV and every 5 s a 250 ms long ramp from −100 mV to +60 mV was performed. Only cells that had a stable resting membrane potential between −50 mV (K^+^ equilibrium) and −35 mV were taken into consideration. Cells were illuminated alternating with UV light (385 nm, 1 mW/mm^2^) and green light (500–600 nm, 14 mW/mm^2^) for 75 s each. Illumination was performed with a 385 nm LED and a 500–600 nm LED within the LEDHub and reflected through a light guide (2 mm, 0.5 NA), dual port condenser (TILL Photonics) and a T580 LPXR (F48-580) dichroic mirror onto the cells. De- or activation of GIRK channels was calculated by dividing the currents at −80 mV during UV light illumination by those during green light illumination. G_q_ proteins were blocked by applying 1 µM FR900359 for 10 min prior to the illumination protocol and G_i_ proteins were blocked by incubating with 500 ng/mL Pertussis-toxin (Sigma-Aldrich) overnight.

### All optical high throughput screening

For initial experiments (Fig. 4a) transgenic HEK cells expressing hOPN5 and transfected with a TPRC6 expression plasmid or hOPN5 and transfected with an empty pcDNA3 plasmid as control were seeded in 384 well plates with 6,000 cells / well, (#781092, Greiner Bio-One,) in 30 µL test medium 1 (DMEM F12, 10% FCS, 20 mM HEPES, 1.35 mM Na-pyruvate, 1% 100x non-essential amino acids, 1% Pen/Strep, 2% poly D-lysine (all Gibco, Thermo Fisher Scientific)) and incubated at 37 °C, 5% CO_2_ for 24 h. Subsequently, medium was exchanged with 30 µL assay buffer 1 (Ca^2+^ free Tyrode (PAN Biotech, Germany) and addition of 2 mM CaCl_2_, 2 µM bis(trifluoromethyl)pyrazoles2 (BTP2, Calbiochem, Sigma-Aldrich), 10 µM cyclopiazonic acid (CPA), 0.2 mg/mL brilliant black) supplemented with 6.3 mM probenecid, 0.01% pluronic and 120 µM Fluo-8 and incubated at 37 °C, 5% CO_2_ for 45 min. hOPN5 was activated by illuminating the plates with UV-LEDs (370/60 nm excitation filter; 31 µW/mm^2^). Fluorescence was measured using blue LEDs (475/35 nm excitation filter, 536/40 nm emission filter) and an ImagEM X2 Camera (Hamamatsu Photonics, Japan).

For further assay optimization (Fig. 4b, c) transgenic HEK cells expressing GCaMP6, hOPN5 and TRPC6 and for compound validation (Fig. 4e, f) transgenic HEK cells expressing GCaMP6 and TRPC6 (TRPC6 assay) or GCaMP6 alone (mAChR assay) were seeded in 384 well plates (5000 cells/well) in 30 µL test medium 1 and incubated at 37 °C, 5% CO_2_ for 24 h. Subsequently, medium was exchanged with 30 µL assay buffer 1 or in the case of HEK mock buffer 1 without BTP-2 and CPA and incubated at 37 °C, 5% CO_2_ for 20 min. Compounds were dissolved in DMSO (1 µL) and prediluted with 55 µL assay buffer 2 (Ca^2+^ free Tyrode and addition of 2 mM CaCl_2_, 0,01% BSA). In all, 10 µL of prediluted compound solution was added to the cells and further incubated at 30 °C, 5% CO_2_ for 10 min. Cells were activated either by addition of ACh (100 µM final concentration) in assay buffer 2 lacking BSA or by illuminating the plates with UV-LEDs (370/60 nm excitation filter; 24 µW/mm^2^). GCaMP6 fluorescence was measured using blue LEDs (475/35 ex, 17 µW/mm^2^, 536/40 em).

For high throughput screening (HTS, Fig. 4d), transgenic HEK GCaMP6 TRPC6 hOPN5 cells were seeded in 1536 well plates (#782092, Greiner Bio-One) with 1000 cells/well, in 7 µL test medium 2 (DMEM F12, 10% FCS, 20 mM HEPES, 1.35 mM Na-pyruvate, 1% 100x non-essential amino acids, 1% Pen/Strep, 4 mM GlutaMax, 2% sodium bicarbonate) and incubated at 37 °C, 5% CO_2_ for 48 h. Subsequently, 5 µL medium was exchanged with 5 µL assay buffer 1 and incubated at 37 °C, 5% CO_2_ for 12 min. Compounds were dissolved in DMSO (0.5 µL, 2 mM) and prediluted with 8 µL assay buffer 2. 0.3 µL of prediluted compound solution was added to the cells and further incubated at 30 °C, 5% CO_2_ for 11 min. hOPN5 was activated by UV-LEDs (370/60 nm excitation filter; 5.2 µW/mm^2^) and endogenous muscarinic receptors by ACh (100 µM final concentration). GCaMP6 fluorescence was measured using blue LEDs (475/35 ex, 11 µW/mm^2^, 536/40 em).

Fluorescence F was normalized to F_0_ at the start of the measurement and background was subtracted resulting in ΔF/F_0_ for further analysis.

### Frequency analysis of ES cell-derived cardiomyocytes

R1 mouse embryonic stem cells (ES cells) were cultured as previously described^21^ on neomycin-resistant mouse embryonic fibroblasts in high-glucose Dulbecco’s Modified Eagle’s Medium (KnockOut DMEM, Invitrogen, U.S.) containing 15% FCS (Capricorn Scientific, Germany), 0.1 mM nonessential amino acids, 100 U/mL penicillin, 100 mg/mL streptomycin, 2 mg/mL l-glutamine (Invitrogen), 0.1 mM β-mercaptoethanol (Sigma-Aldrich) and 500 U/mL leukemia inhibitory factor (Chemicon, U.S.). DNA transfection was performed by electroporation (250 V, 500 µF, Gene Pulser Xcell, Bio-Rad, U.S.) of 4 × 10^6^ ES cells in PBS (Invitrogen) mixed with 40 µg of the linearized CAG hOPN5 eYFP plasmid. Afterwards, ES cells were plated and antibiotic selection with 300 µg/mL G418 was started 24 h after transfection. eYFP positive clones were picked and further propagated. Differentiation was achieved within hanging drops containing 400 ES cells in IMDM with 20% FCS supplemented with 100 U/mL penicillin and 100 mg/mL streptomycin for two days to generate embryoid bodies (EBs). EBs were washed, cultured in suspension on a horizontal shaker for three days, plated on 0.1% gelatin-coated glass cover slips and started to beat spontaneously after day 7 of differentiation. Prior experiments, cells were incubated at 37 °C in fresh 20% IMDM with 2 µM ATR for two hours. During experiments, EBs were perfused continuously with Tyrode solution (see above) at 35 °C. The beating was recorded on an Axiovert 200 microscope through a 5x objective (Fluar 5x, Zeiss) with a piA640-210gm camera (Basler, Germany) at 50 frames per second and analyzed online using a custom-made software as reported previously^21^. Beating was imaged with infrared light (760 nm) to avoid hOPN5 activation. UV light (385 nm) was generated within the LEDHub and coupled into the microscope as described above and reflected onto the specimen via T580 LPXR (F48-580) and a 594 nm LP Edge Basic LP long pass filter (AHF Analysetechnik). Pharmacological stimulation was performed with Endothelin 1 (ET-1, Sigma-Aldrich) and G_q_ proteins were blocked with 1 µM FR900359.

### Generation of transgenic mice and heart weight measurements

hOPN5/eYFP transgenic mice were created by pronuclear injection of FVB/N mice using standard procedures by the core facility of the Max-Planck Institute of Experimental Medicine, Göttingen. The part comprising the CAG promoter, the hOPN5/eYFP gene as well as the BGH PolyA sequence were excised with MfeI and DraIII and purified with the Freeze ‘N Squeeze DNA Gel Extraction kit (Bio-Rad) and MinEluteR PCR purification kit (#28004, Qiagen, Germany). Eight founder mice and their resulting heterozygous offsprings were genotyped by a standard polymerase chain reaction using the primers against eYFP: 5′-CATGAAGCAGCACGACTTCT-3′ and 5′-GCGGATCTTGAAGTTCACCT-3′, resulting in a 274-bp fragment. Positive founder mice were mated with CD1 wild-type animals to obtain heterozygous offspring and wild-type littermate controls. We obtained two founder lines with high expression rate of hOPN5/eYFP in the heart, skeletal muscle and organs with smooth muscle cells. Including all experiments, we used 34 male and 29 female transgenic OPN5 mice (16 ± 1.1 weeks old), 31 male and 15 female wild-type siblings (12 ± 0.85 weeks old) and 15 male and 18 female CD1 wild-type mice (27 ± 2.5 weeks old) as controls. Mice were killed by cervical dislocation and experiments were performed with explanted organs. Hearts were explanted, perfused with DPBS solution (Sigma-Aldrich) and dried for 5 h at 37 °C to determine dry heart weight normalized to the femur length of mice at 2 months age (±7 days).

Melanopsin expressing transgenic mice were generated using a previously described G4 embryonic stem cell line stably transfected with the CAG-melanopsin-IRES-GFP plasmid^22^. Aggregation of ES cells with 40 chromosome karyotype and diploid morula stage CD1 embryos was performed as reported before. Chimeric mice were identified by their coat color chimerism and bred to CD1 mice to test germline transmission. Transgene expression in offspring with agouti coat color was verified by detection of GFP signal in tail tissue. The mice investigated in this study were back-crossed at least 10 generations. Mice of this transgenic mouse line were in total 2 male and 3 female (51 ± 4.2 weeks old) and 5 female CD1 wild-type mice (20 ± 1.1 weeks old) as controls.

### Stimulation of explanted hearts ex vivo

Mouse hearts were explanted after cervical dislocation and immediately placed in ice-cold DPBS. Surrounding tissue was dissected, and hearts were retrogradely perfused via the aorta with Tyrode’s solution (see above) containing 1 µM ATR. A bipolar cardiac electrogram was obtained using a silver electrode in contact with the right atrium and a metal spoon placed under the heart’s apex. The signal was amplified with an animal bio-amplifier (Animal Bio Amp FE136, AD Instruments) and a PowerLab 16/35 recording system and analyzed with LabChart 8.1.16 software. The heart was placed below a macroscope (MVX10, MVLPAPO1x, NA: 0.25, Olympus) connected with a LEDHub through a light-guide (Ø 2 mm, NA 0.5). The eYFP phenotype was confirmed using the 500–600 nm LED and the F46-003XL filterset (AHF Analysetechnik). Illumination of the right atrial posterior surface at the junction of the superior vena cava was performed using the 385 nm UV-LED and the light intensity (light power divided by the illuminated area in mW/mm^2^) was calibrated with an optical power meter (PM100 and detector S170C Thorlabs, USA).

After the establishment of regular sinus rhythm, for hOPN5 hearts, an illumination protocol was performed consisting of three light pulses of 10 s duration, a light intensity of 1 mW/mm^2^, with at least 2 min delay in-between. The increase in heart rate was analyzed by averaging seven subsequent sinus beats before illumination and around the peak of heart rate increase after illumination. Premature extrasystoles were excluded from analysis. For statistical analysis we took the average increase in heart rate per heart of all three illuminations into consideration.

Melanopsin hearts were illuminated with a 460 nm LED within the LEDHub focused to a spot size of 36 mm^2^ within the posterior wall of the right atrium. Light was applied for 1 s every 4 min (1.4 mW/mm^2^), calibrated by a power meter (PM100 and detector S130A, Thorlabs). RR interval traces were smoothed by averaging of 1 s intervals and the normalized frequency change was defined as the minimum frequency 30 s after illumination relative to the minimum frequency 30 s before illumination.

### Isolation of adult ventricular cardiomyocytes

Cardiomyocytes were isolated according to earlier reports^73^. Briefly, explanted hearts were retrogradely perfused in a Langendorff configuration with perfusion buffer (in mM: NaCl 113, KCl 4.7, KH_2_PO_2_ 0.6, Na_2_HPO_4_ 0.6, MgSO_4_·7H_2_O 1.2, Phenol red 0.032, NaHCO_3_ 12, KHCO_3_ 10, HEPES buffer 10, Taurine 30, 2,3 BDM 10, Glucose 10; pH adjusted to 7.2 using NaOH) for 5 min at 37 °C and subsequently with digestion buffer (perfusion buffer, CaCl_2_ 12.5 µM, Liberase DH (Sigma-Aldrich) 0.06 mg/mL, Trypsin 0.14 mg/mL) for 14–16 min (if not stated otherwise all drugs from Sigma-Aldrich). Ventricles were transferred into a 60 mm petri dish containing 2.5 mL digestion buffer and dispersed using two forceps. Stop buffer (perfusion buffer with BSA 10%) was added to the mixture which was then filtered through a 200 µm mesh. Finally, Ca^2+^ was re-introduced in four steps to 1.2 mM over 20 min and cardiomyocytes were plated on Laminin (10 µg/mL, Sigma-Aldrich) coated coverslips and incubated for at least 1 h at 37 °C and 5% CO_2_.

To analyze the expression rate of hOPN5/eYFP, pictures of dissociated adult cardiomyocytes were taken using a IX73 fluorescence microscope with 10 × objective UPLFLN10X2PH NA 0.3, Olympus), the 500–600 nm LED of the LedHUB and F46-003 filterset (AHF Analysetechnik). Electrical stimulation (1 Hz, 2 ms long biphasic pulses, 46 V) was performed with two platinum electrodes and the Myostim stimulator (Myotronic, Germany) and illumination with UV light (1 mW/mm², 100 ms) using the 385 nm LED within the LedHub.

To analyze the contractility of the isolated adult cardiomyocytes, we designed a custom program implementing Farneback’s dense optical flow algorithm^74^. Briefly, a live feed of one cardiomyocyte was captured with an inverted IX73 microscope and the 20x objective (LUCPLFLN20XPH/0,45) using a UI-306xCP-M camera (iDS, Germany), and the displacement motion vectors were calculated by approximating movements of pixels from two subsequent frames (f_0_ and f_1_) using a quadratic polynomial function:

$${{{{{{\rm{f}}}}}}}_{0} \sim {{{{{{\rm{x}}}}}}}^{{{{{{\rm{T}}}}}}}{{{{{{\rm{A}}}}}}}_{0}{{{{{\rm{x}}}}}}+{{{{{{{\rm{b}}}}}}}_{0}}^{{{{{{\rm{T}}}}}}}{{{{{\rm{x}}}}}}+{{{{{{\rm{c}}}}}}}_{0}$$

$${{{{{{\rm{f}}}}}}}_{1} \sim {{{{{{\rm{x}}}}}}}^{{{{{{\rm{T}}}}}}}{{{{{{\rm{A}}}}}}}_{1}{{{{{\rm{x}}}}}}+{{{{{{{\rm{b}}}}}}}_{1}}^{{{{{{\rm{T}}}}}}}{{{{{\rm{x}}}}}}+{{{{{{\rm{c}}}}}}}_{1}$$

Subsequently, the absolute average of motion vectors per frame |V| was outputted for online analysis to LabChart 8.1.16 software through NI 9263 CompactDAQ (NI, Austin, U.S.). The code for the myocyte online contraction analysis can be found at https://github.com/awagdi0/MOCA/.

### Isometric force measurements of smooth muscle function

In total, 7–10-mm-long specimen of the small intestine, strips from the bladder as well as uterine horns were harvested from wild type and hOPN5 transgenic mice. Small intestine specimens were immediately flushed to clean the lumen of remaining food and digestive secretions. Samples were kept at 4 °C and used within 24 h. For isometric force measurements, small intestine specimen and uteri were tied to a TR5S optical force sensor (Myotronic, Germany) or FT20 force transducer (Hugo Sachs Elektronik, Germany) and submerged in a horizontal 5 ml or vertical 20 ml organ bath containing 2 opposing platin electrodes capable of electric field stimulation (EFS). Data were recorded by a PowerLab 8/35 and the LabChart 8.1.16 software. The organ bath was filled with Krebs solution (in mM: NaCl 112, KCl 4.7, CaCl_2_ 2.5, MgCl_2_ 1.2, Glucose 11.5, KH_2_PO_4_ 1.2, NaHCO_3_ 25) pre-heated to 37 °C, gassed with carbogen (95% O_2_ / 5% CO_2_) and supplemented with 5 µM ATR. Specimen were given at least 15 min time to equilibrate and develop rhythmic, endogenous contraction patterns and adjusted to a pre-stretch of up to 11 mN at the beginning of measurements.

For uteri, 5 mM MgCl_2_ was added to the bath to prevent intrinsic contractions. Subsequently, samples were stimulated by EFS as well as UV light. Electrical stimulation was achieved by applying 100 Hz trains of 100 pulses for small intestine specimen and 300 pulses for uteri, with a pulse width of 2 ms and a pulse height of 40 V, using the Myostim stimulator. Illumination was performed with a 385 nm LED within the LEDHub and transmitting the light via a 2 mm light guide (NA 0.5) which illuminated the sample orthogonally through the glass wall of the organ bath. For UV light stimulation, light impulses with a duration of 2 s for small intestine specimen or 5 s for uteri were applied at a light intensity of 3 mW/mm.^2^ To investigate dependency of isometric force on light intensity in small intestine specimen, samples were stimulated with 2 s long light pulses of increasing intensities of 1, 3, 10, 30, 100, 300, 1000, and 3000 µW/mm^2^ in intervals of 2 min. The relation between isometric force and illumination duration was analyzed by applying light pulses of 3 mW/mm² intensity and increasing pulse durations of 1, 3, 10, 30, 100, 300, 1000, and 3000 ms in intervals of 2 min. Finally, stimulation of both small intestine specimen and uteri was concluded by addition of 10 µM CCh or 60 mM KCl, while exchanging the bath with fresh Krebs solution in between and recovering periods of at least 5 min to the respective stimulus. For CCh and KCl stimulation, responses were measured as differences between maximum force after and before substance application. Responses to light and electrical field stimulation were quantified as maximal force between initiation and 10 s after termination of the respective stimulus.

To evaluate at which pulse interval subsequent pulses would increase the maximum generated isometric force, specimen were illuminated with 10 s long illumination patterns with decreasing pulse intervals (10 s, 3.3 s, 2 s, 1 s, 0.33 s, 0.1 s) at a given pulse length (10 ms, 30 ms, 100 ms, 300 ms). The maximum isometric force was normalized to the maximum force induced by 10 s long continuous illuminations. Furthermore, the relationship between isometric force and the duty cycle of pulsed illumination was investigated by illuminating with 10 s long illumination patterns with varying frequency (0.3 Hz, 1 Hz, 3 Hz, 10 Hz) and decreasing pulse length stepwise. Isometric force was quantified as maximal force between initiation and termination of the respective illumination pattern minus the average cyclic height of 1 min of endogenous contraction pattern prior to illumination. Half maximal duty cycle was investigated by fitting the values of each specimen with a Hill fit. The desensitization and recovery time of light-induced contractions was quantified by applying two subsequent UV light pulses with the same pulse duration (3 s, 10 s, 30 s, 1 mW/mm^2^) but with varying interpulse intervals (single pulse, 0.01 s, 1 s, 3 s, 5 s, 10 s, 20 s, 40 s, 80 s). An interval of at least 4 min was kept between each pulse pair. The maximal isometric force of the second peak was normalized to the respective first peak. To analyse the recovery time, the values of each specimen were fitted with a one phase decay fit and half maximal recovery time was compared between the different pulse lengths. Only fits with a *R*^2^ values >0.7 were included in the analysis.

Bladder strips were tethered to a glass holder and mounted in 30 ml vertical organ baths filled with Krebs solution at 37 °C and gassed with carbogen and pre-stretched to 2–3 mN. Isometric force was measured with MLT0201 transducers (PanLab Havard Apparatus, USA) connected to quad bridge amplifiers and a PowerLab 16/35 using the LabChart 8.1.16 software (all ADinstruments, Australia). After at least 20 min equilibration, samples were stimulated by UV light using 10 s long pulses. Illumination was performed with a LEDHub equipped with a 385 nm LED and transmitting the light via a 2 mm light guide (NA 0.5) which illuminated the sample orthogonally through the glass walling of the organ bath. Bladders were furthermore stimulated by addition of 60 mM KCl or 10 µM CCh, while exchanging the bath with fresh Krebs solution in between and a recovering period of at least 5 min to the respective aforegoing stimulus. For CCh and KCl stimulation, responses were measured as differences between maximum force after and before substance application. Responses to light and electrical field stimulation were quantified as maximal force between initiation and 10 s after termination of the respective stimulus.

### Histology

Isolated adult cardiomyocytes were fixed using 4% formaldehyde for 20 min and then washed with DPBS and permeabilised with 0.2% Triton X100 for 20 min. Subsequently coverslips were stained in DPBS supplemented with 5% donkey serum for 2 h at room temperature and primary antibodies against GFP (3H9-100, Chromotek, Germany, 1:800), and α-Actinin (A7811, Sigma-Aldrich, 1:400). Staining with secondary antibodies conjugated with Cy2 and Cy5 (712-225-153, 712-225-152, JacksonLab, U.S., 1:400) diluted in DPBS with 1:1000 DAPI (0018860.01, Th.Geyer,) was performed for 1 h at room temperature. Pictures of single cardiomyocytes were taken with an IX83 inverted fluorescence microscope equipped with an ORCA-flash 4.0 digital camera (C11440, Hamamatsu Photonics) and the MT20 illumination system as light source controlled via the CellSens® software. Acquisition of images was performed with a 60x objective (UPLSAPO60X, NA: 1.35) with following filter settings: 387/11 excitation, 410 beamsplitter and 440/40 emission for DAPI, 485/20, 504 and 525/30 for eYFP, 560/25, 582, and 684/24 for Cy5.

Embryonic stem cells (ESC) and ESC-derived cardiomyocytes were fixed with 4% formaldehyde for 20 min and later stained with the stem cell marker Oct3/4 (Santa Cruz Biotechnology, sc-9081, 1:100) and with anti-α-actinin (Sigma Aldrich, A7811, 1:400), respectively, diluted in DPBS with 0.2% Triton X100 and 5% donkey serum for 2 h at room temperature. Alexa-Fluor 647 goat anti-rabbit (Thermofisher Scientific, A-31573), IgG or Alexa Fluor 647 goat anti mouse IgG1 (Thermo Fisher Scientific, A-21240) secondary antibodies were applied at a dilution of 1:400 for one hour at room temperature in a solution with 1 µg/mL Hoechst 33342 (Thermo Fisher Scientific). Immunostainings were documented with a microscope Observer Z1 with ApoTome (Zeiss).

For confocal images of ventricular tissue, explanted hearts were sliced (1–3 mm thick slices) and fixed using 4% formaldehyde for 4 h, then underwent the PEGASOS clearing protocol^75^ and stained with primary antibodies against GFP (Chromotek, 3H9-100, 1:400) and α-Actinin (Sigma Aldrich, A7811, 1:400) as well as secondary antibodies conjugated with Cy2, and Cy3 (JacksonLab, 712-225-153, 712-225-152, 1:400) and DAPI (1:1000) for 7 days at 37 °C. For small intestine images, explanted specimen were fixed using 4% formaldehyde for 24 h and then transferred to PBS containing 20% saccharose for 48 h. The samples were cryopreserved with Tissue-Tek (Sakura, Germany) and sectioned with a HM560 cryotome (Thermo Scientific, Germany) into slices of 8 µm thickness. Subsequent permeabilization was performed with 0.2% TritonX for 20 min. Slices were stained in PBS supplemented with 5% donkey serum for 2 h at room temperature with primary antibodies against GFP (Sigma-Aldrich 11.814.460.001, 1:800), β-III-Tubulin (BioLegend 802001, 1:800) and c-Kit (Linaris MAK5302, 1:400). Staining with secondary antibodies conjugated with Cy2 (1:200, 715-225-151), Cy3 (1:400, 711-165-152), or Cy5 (1:400, 712-175-153, JacksonLab, USA) diluted in PBS with 1:1000 DAPI was performed for 1 h at room temperature.

Images of ventricular and small intestine slices were taken using a LSM 800 confocal microscope (Zeiss) equipped with spectral multi-alkali photomultiplier detectors using a 63x objective (LCI Plan-Neofluar 63X, NA: 1.3) via the ZEN 2.6 (blue edition) software (Zeiss) with a pinhole of 1 AU (ventricular slice: 46 µm for Cy2, 255 µm for Cy5, and 42 µm for DAPI, small intestine: (46 µm for Cy2, 53 µm for Cy3, 255 µm for Cy5 and 48 µm for DAPI).

For whole-heart 3D images, hearts were fixed with 4% formaldehyde for 24 h. Subsequently hearts were washed with DPBS and underwent the PEGASOS clearing protocol^75^ and stained for 14 days at 37 °C with primary antibodies against GFP (1:100, Chromotek, 3H9-100), and HCN4 (APC-052, Alomone, Israel, 1:200) and subsequently with secondary antibodies conjugated with Cy2 (712-225-153, 1:200), and Cy5 (712-225-152, 1:200, JacksonLab) for 14 days at 37 °C. Images were acquired using the light sheet microscope UltraMicroscope II (LaVision, Germany) equipped with an Andor NEO camera (Oxford Instruments) and SuperK Extreme EXW12 as a light source (NKT Photonnics, Denmark) using the following filter settings: 470/30ex, and 525/50em for Cy2, 630/30ex, and 680/30em for Cy5, and deconvoluted^76^. 3D models were reconstructed using Imaris x64 9.6.0 (Oxford Instruments).

### Statistics and reproducibility

Statistical data are shown as mean ± standard error of the mean and were analyzed with the GraphPad Prism 8 software. Each dot represents an independent experiment (number of each represented by *n*, number of mice reported as *N*). Statistical analysis was performed with an ordinary one-way ANOVA test with a Tukey multiple comparison test for the comparison of hearts of the different mouse lines, contractility changes in adult cardiomyocytes, ecg parameters, half maximal duty cycles, OPN5 TRPC6 and only OPN5 cells as well as a repeated measurements one-way ANOVA with Geisser-Greenhouse correction and Tukey´s multiple comparison test for analysis of the maximal desensitization and the recovery time in dependence of the pulse duration, a two-way repeated measurements ANOVA and Sidak’s multiple comparison to test the effect of hOPN5 vs wild-type control and Tukey’s multiple comparison to test between different treatments within the hOPN5 group as well as the effects in KO cells compared HEK wild-type cells. Effects in melanopsin expressing hearts compared to wild-type controls, validation of the HTS compounds and comparisons between HEK hOPN5/eYFP to HEK hOPN5 IRES eGFP cells were analyzed with an unpaired two-sided Student’s *t* test. UV light effects on the spontaneous beating rate in ES cell-derived cardiomyocytes with a paired two-sided Student’s *t* test. *P* < 0.05 was considered statistically significant and significances are indicated as * for *P* ≤ 0.05, **P ≤ 0.01, ***P ≤ 0.001 and ****P ≤ 0.0001. All pictures are taken from representative experiments and images (*n* ≥ 3).

### Reporting summary

Further information on research design is available in the Nature Research Reporting Summary linked to this article.

## Supplementary information

Supplementary Information

Reporting Summary

Description of Additional Supplementary Files

Supplementary Movie 1

Supplementary Movie 2

Supplementary Movie 3

## Data Availability

All data points shown in figures for statistical analysis are presented in the Source Data file. Data from the HTS (Fig. 4d) are also provided in the Source Data file in an anonymized format because exact substance names and structures are proprietary to Bayer AG and cannot be provided. Source data are provided with this paper.

## Source data

Source Data

## Code availability

The code for the myocyte online contraction analysis can be found at https://github.com/awagdi0/MOCA/.

## References

[1] Neves SR, Ram PT, Iyengar R (2002). G protein pathways. Science.

[2] Kamato D (2017). Gaq proteins: molecular pharmacology and therapeutic potential. Cell. Mol. Life Sci..

[3] Sriram K, Insel PA (2018). G protein-coupled receptors as targets for approved drugs: how many targets and how many drugs?. Mol. Pharmacol..

[4] Wettschureck N, Offermanns S (2005). Mammalian G proteins and their cell type specific functions. Physiol. Rev..

[5] Kleinlogel S (2016). Optogenetic user’s guide to Opto-GPCRs. Front. Biosci..

[6] Lyon AM, Taylor VG, Tesmer JJ (2014). Strike a pose: Galphaq complexes at the membrane. Trends Pharmacol. Sci..

[7] Masseck OA, Rubelowski JM, Spoida K, Herlitze S (2011). Light- and drug-activated G-protein-coupled receptors to control intracellular signalling. Exp. Physiol..

[8] Sharif-Naeini R (2010). Sensing pressure in the cardiovascular system: Gq-coupled mechanoreceptors and TRP channels. J. Mol. Cell. Cardiol..

[9] Sanchez-Fernandez G (2014). Galphaq signalling: the new and the old. Cell Signal..

[10] Hubbard KB, Hepler JR (2006). Cell signalling diversity of the Gqalpha family of heterotrimeric G proteins. Cell Signal..

[11] Offermanns S, Simon MI (1998). Genetic analysis of mammalian G-protein signalling. Oncogene.

[12] Zhang L, Shi G (2016). Gq-coupled receptors in autoimmunity. J. Immunol. Res..

[13] Kostenis E, Pfeil EM, Annala S (2020). Heterotrimeric Gq proteins as therapeutic targets?. J. Biol. Chem..

[14] Chua V (2017). Dysregulated GPCR signaling and therapeutic options in uveal melanoma. Mol. Cancer Res..

[15] Kirschenbaum MA, Serros ER, Lowe A (1979). Variability in rates of urine prostaglandin E excretion. Prostaglandins.

[16] Mederos YSM, Storch U, Gudermann T (2016). Mechanosensitive Gq/11 protein-coupled receptors mediate myogenic vasoconstriction. Microcirculation.

[17] Wang W, Qiao Y, Li Z (2018). New insights into modes of GPCR activation. Trends Pharmacol. Sci..

[18] Karunarathne WK, Giri L, Kalyanaraman V, Gautam N (2013). Optically triggering spatiotemporally confined GPCR activity in a cell and programming neurite initiation and extension. Proc. Natl Acad. Sci. USA.

[19] Spoida K (2016). Melanopsin variants as intrinsic optogenetic on and off switches for transient versus sustained activation of G protein pathways. Curr. Biol..

[20] Bailes HJ (2017). Optogenetic interrogation reveals separable G-protein-dependent and -independent signalling linking G-protein-coupled receptors to the circadian oscillator. BMC Biol..

[21] Makowka P (2019). Optogenetic stimulation of Gs-signaling in the heart with high spatio-temporal precision. Nat. Commun..

[22] Beiert T, Bruegmann T, Sasse P (2014). Optogenetic activation of Gq signalling modulates pacemaker activity of cardiomyocytes. Cardiovasc. Res..

[23] van Wyk M, Pielecka-Fortuna J, Lowel S, Kleinlogel S (2015). Restoring the ON switch in blind retinas: opto-mGluR6, a next-generation, cell-tailored optogenetic tool. PLoS Biol..

[24] Simon CJ, Sahel JA, Duebel J, Herlitze S, Dalkara D (2020). Opsins for vision restoration. Biochem. Biophys. Res. Commun..

[25] Ferrari U (2020). Towards optogenetic vision restoration with high resolution. PLoS Comput. Biol..

[26] Oh E, Maejima T, Liu C, Deneris E, Herlitze S (2010). Substitution of 5-HT1A receptor signaling by a light-activated G protein-coupled receptor. J. Biol. Chem..

[27] Masseck OA (2014). Vertebrate cone opsins enable sustained and highly sensitive rapid control of Gi/o signaling in anxiety circuitry. Neuron.

[28] Gutierrez DV (2011). Optogenetic control of motor coordination by Gi/o protein-coupled vertebrate rhodopsin in cerebellar Purkinje cells. J. Biol. Chem..

[29] Eickelbeck D (2019). CaMello-XR enables visualization and optogenetic control of Gq/11 signals and receptor trafficking in GPCR-specific domains. Commun. Biol..

[30] Bailes HJ, Zhuang LY, Lucas RJ (2012). Reproducible and sustained regulation of Galphas signalling using a metazoan opsin as an optogenetic tool. PLoS ONE.

[31] Bailes HJ, Lucas RJ (2013). Human melanopsin forms a pigment maximally sensitive to blue light (lambdamax approximately 479 nm) supporting activation of G(q/11) and G(i/o) signalling cascades. Proc. Biol. Sci..

[32] Ye H, Daoud-El Baba M, Peng RW, Fussenegger M (2011). A synthetic optogenetic transcription device enhances blood-glucose homeostasis in mice. Science.

[33] Airan RD, Thompson KR, Fenno LE, Bernstein H, Deisseroth K (2009). Temporally precise in vivo control of intracellular signalling. Nature.

[34] Yamashita T (2014). Evolution of mammalian Opn5 as a specialized UV-absorbing pigment by a single amino acid mutation. J. Biol. Chem..

[35] Yamashita T (2010). Opn5 is a UV-sensitive bistable pigment that couples with Gi subtype of G protein. Proc. Natl Acad. Sci. USA.

[36] Tarttelin EE, Bellingham J, Hankins MW, Foster RG, Lucas RJ (2003). Neuropsin (Opn5): a novel opsin identified in mammalian neural tissue. FEBS Lett..

[37] Nakane Y, Shimmura T, Abe H, Yoshimura T (2014). Intrinsic photosensitivity of a deep brain photoreceptor. Curr. Biol..

[38] Nakane Y (2010). A mammalian neural tissue opsin (Opsin 5) is a deep brain photoreceptor in birds. Proc. Natl Acad. Sci. USA.

[39] Kojima D (2011). UV-sensitive photoreceptor protein OPN5 in humans and mice. PLoS ONE.

[40] Haltaufderhyde K, Ozdeslik RN, Wicks NL, Najera JA, Oancea E (2015). Opsin expression in human epidermal skin. Photochem. Photobiol..

[41] Buhr ED (2015). Neuropsin (OPN5)-mediated photoentrainment of local circadian oscillators in mammalian retina and cornea. Proc. Natl Acad. Sci. USA.

[42] Buhr ED, Vemaraju S, Diaz N, Lang RA, Van Gelder RN (2019). Neuropsin (OPN5) mediates local light-dependent induction of circadian clock genes and circadian photoentrainment in exposed murine skin. Curr. Biol..

[43] Zhang KX (2020). Violet-light suppression of thermogenesis by opsin 5 hypothalamic neurons. Nature.

[44] Sugiyama T, Suzuki H, Takahashi T (2014). Light-induced rapid Ca(2)(+) response and MAPK phosphorylation in the cells heterologously expressing human OPN5. Sci. Rep..

[45] Schrage R (2015). The experimental power of FR900359 to study Gq-regulated biological processes. Nat. Commun..

[46] Hisano Y (2019). Lysolipid receptor cross-talk regulates lymphatic endothelial junctions in lymph nodes. J. Exp. Med..

[47] Tennigkeit SA (2019). Design of an ultrafast G protein switch based on a mouse melanopsin variant. Chembiochem.

[48] Breitwieser GE (2005). GIRK channels: hierarchy of control. Focus on “PKC-delta sensitizes Kir3.1/3.2 channels to changes in membrane phospholipid levels after M3 receptor activation in HEK-293 cells”. Am. J. Physiol. Cell Physiol..

[49] Inglese J (2007). High-throughput screening assays for the identification of chemical probes. Nat. Chem. Biol..

[50] Maier T (2015). Discovery and pharmacological characterization of a novel potent inhibitor of diacylglycerol-sensitive TRPC cation channels. Br. J. Pharmacol..

[51] Dahlin JL (2015). PAINS in the assay: chemical mechanisms of assay interference and promiscuous enzymatic inhibition observed during a sulfhydryl-scavenging HTS. J. Med. Chem..

[52] Bruegmann T (2010). Optogenetic control of heart muscle in vitro and in vivo. Nat. Methods.

[53] Mery A (2005). Initiation of embryonic cardiac pacemaker activity by inositol 1,4,5-trisphosphate-dependent calcium signaling. Mol. Biol. Cell.

[54] Mika, D. & Fischmeister, R. Cyclic nucleotide signaling and pacemaker activity. *Prog. B**iophys. Mol. Biol.* **166**, 29–38 (2021). 10.1016/j.pbiomolbio.2021.07.007 34298001

[55] MacDonald EA, Rose RA, Quinn TA (2020). Neurohumoral control of sinoatrial node activity and heart rate: insight from experimental models and findings from humans. Front. Physiol..

[56] Ju YK (2011). Distribution and functional role of inositol 1,4,5-trisphosphate receptors in mouse sinoatrial node. Circ. Res..

[57] Mishra S (2010). Cardiac hypertrophy and heart failure development through Gq and CaM kinase II signaling. J. Cardiovasc. Pharmacol..

[58] Sunahara RK, Taussig R (2002). Isoforms of mammalian adenylyl cyclase: multiplicities of signaling. Mol. Interv..

[59] Atwood BK, Lopez J, Wager-Miller J, Mackie K, Straiker A (2011). Expression of G protein-coupled receptors and related proteins in HEK293, AtT20, BV2, and N18 cell lines as revealed by microarray analysis. BMC Genomics.

[60] Lan Y, Zeng W, Dong X, Lu H (2021). Opsin 5 is a key regulator of ultraviolet radiation induced melanogenesis in human epidermal melanocytes. Br. J. Dermatol..

[61] Amobi NI, Smith IC (1987). Adrenergic and ‘non-adrenergic’ contributions to the two-component tetanus in the rat vas deferens. Eur. J. Pharmacol..

[62] Laekeman GM, Herman AG (1978). Prostaglandins restore the hyoscine-induced inhibition of the guinea-pig ileum. Prostaglandins.

[63] Sjoblom N, Nilsson H, Folkow B (1987). Post-tetanic potentiation of the vascular neurogenic response in rats. Acta Physiol. Scand..

[64] Mattis J (2011). Principles for applying optogenetic tools derived from direct comparative analysis of microbial opsins. Nat. Methods.

[65] Wietek J (2017). Anion-conducting channelrhodopsins with tuned spectra and modified kinetics engineered for optogenetic manipulation of behavior. Sci. Rep..

[66] Berry MH (2019). Restoration of high-sensitivity and adapting vision with a cone opsin. Nat. Commun..

[67] Gundelach LA, Huser MA, Beutner D, Ruther P, Bruegmann T (2020). Towards the clinical translation of optogenetic skeletal muscle stimulation. Pflug. Arch..

[68] Vogt M (2021). Direct optogenetic stimulation of smooth muscle cells to control gastric contractility. Theranostics.

[69] Jang TM (2020). Expandable and implantable bioelectronic complex for analyzing and regulating real-time activity of the urinary bladder. Sci. Adv..

[70] Maier-Peuschel M (2010). A fluorescence resonance energy transfer-based M2 muscarinic receptor sensor reveals rapid kinetics of allosteric modulation. J. Biol. Chem..

[71] Kaufmann K (2013). ML297 (VU0456810), the first potent and selective activator of the GIRK potassium channel, displays antiepileptic properties in mice. ACS Chem. Neurosci..

[72] Richter F (2015). Upgrading a microplate reader for photobiology and all-optical experiments. Photochem. Photobiol. Sci..

[73] O’Connell TD, Rodrigo MC, Simpson PC (2007). Isolation and culture of adult mouse cardiac myocytes. Methods Mol. Biol..

[74] Farnebäck, G. Two-Frame Motion Estimation Based on Polynomial Expansion. (eds Bigun, J., Gustavsson, T.) In *Image Analysis. SCIA 2003. Lecture Notes in Computer Science*. vol 2749. 363–370 (Springer, Berlin, Heidelberg, 2003).

[75] Jing D (2018). Tissue clearing of both hard and soft tissue organs with the PEGASOS method. Cell Res..

[76] Becker K (2019). Deconvolution of light sheet microscopy recordings. Sci. Rep..

