# Author Correction: Selective optogenetic control of G_q_ signaling using human Neuropsin

**DOI:** 10.1038/s41467-022-35612-8

**Published:** 2023-01-04

**Authors:** Ahmed Wagdi, Daniela Malan, Udhayabhaskar Sathyanarayanan, Janosch S. Beauchamp, Markus Vogt, David Zipf, Thomas Beiert, Berivan Mansuroglu, Vanessa Dusend, Mark Meininghaus, Linn Schneider, Bernd Kalthof, J. Simon Wiegert, Gabriele M. König, Evi Kostenis, Robert Patejdl, Philipp Sasse, Tobias Bruegmann

**Affiliations:** 1 Institute for Cardiovascular Physiology, University Medical Center Göttingen, Göttingen, Germany; 2 German Center for Cardiovascular Research (DZHK), Partner site Göttingen, Göttingen, Germany; 3 Institute of Physiology I, Medical Faculty, University of Bonn, Bonn, Germany; 4 Department of Internal Medicine II, University Hospital Bonn, University of Bonn, Bonn, Germany; 5 Research Training Group 1873, University of Bonn, Bonn, Germany; 6 Bayer AG, Research & Development, Pharmaceuticals, 42096 Wuppertal, Germany; 7 Center for Molecular Neurobiology Hamburg, University Medical Center Hamburg-Eppendorf, Hamburg, Germany; 8 Molecular, Cellular and Pharmacobiology Section, Institute of Pharmaceutical Biology, University of Bonn, Bonn, Germany; 9 Oscar-Langendorff Institute of Physiology, Rostock University Medical Center, Rostock, Germany; 10 Cluster of Excellence “Multiscale Bioimaging: from Molecular Machines to Networks of Excitable Cells” (MBExC), University of Göttingen, Göttingen, Germany; 11 Present Address: Department of Cardiology and Pulmonology, University Medical Center Göttingen, Georg August University of Göttingen, Göttingen, Germany

**Keywords:** Drug development, Arrhythmias, Protein design, Optogenetics

Correction to: *Nature Communications* 10.1038/s41467-022-29265-w, published online 01 April 2022

The original version of this Article omitted a contributor, Michael Hesse, in the Acknowledgements section. The Acknowledgements section now reads:

We are grateful to Melanie von Ahlen, Frank Holst, Sören Petzke, Claudia Herzog, Tobias Ewering, Georg Schmidt and Peter Hombach for technical assistance and Patricia Jonatan, Sophie Curio, Shi Runzhu, and Sophia Katharina Ebert for their contribution during the start of this project. We thank Michael Hesse for generating the OPN4 transgenic mice, Asuka Inoue for providing HEK Gi and Gq/11 knockout HEK cells, David Weaver for providing HEK cells expressing GIRK1/2, Carsten Hoffmann for providing the M2-CFP plasmid and the core facility of the Max-Planck Institute of Experimental Medicine (Göttingen) for generating the hOPN5/eYFP mice. This work was supported and funded by Bonfor program of the Medical Faculty Bonn (T.Be, T.Br, and P.S.), the Göttingen Promotionskolleg für Medizinstudierende (Else Kröner-Fresenius-Stiftung 2017_Promotionskolleg.04, D.Z.), the German Centre for Cardiovascular Research (DZHK, doctoral scholarship to A.W.), the Deutsche Forschungsgesellschaft (DFG, German Research Foundation: Germany’s Excellence Strategy (EXC 2067/1-390729940, T.Br): IRTG1826 (200857327, T.Br, U.S., and M.V.); GRK 1873 (214362475, P.S., T.B., V.D., B.M., and E.K.), Priority Program 1926 (315212873 (T.Br) and 315402240 (P.S.)), CRC1002 (193793266, T.Br), SA 1785/7-1 (313904155, P.S.), and SA 1785/9-1 (380524518, P.S.).

This has been corrected in both the PDF and HTML versions of the Article.

