# Supplementary material for: Selective optogenetic control of Gq signaling using human Neuropsin
Source: Nat Commun. 2022 Apr 1;13:1765. doi: 10.1038/s41467-022-29265-w (PMC8975936; doi:10.1038/s41467-022-29265-w)
Supplement: Supplementary file 1 — Supplementary Information [file 41467_2022_29265_MOESM1_ESM.pdf]

Wagdi et al., 2022: Supplementary Figure 1

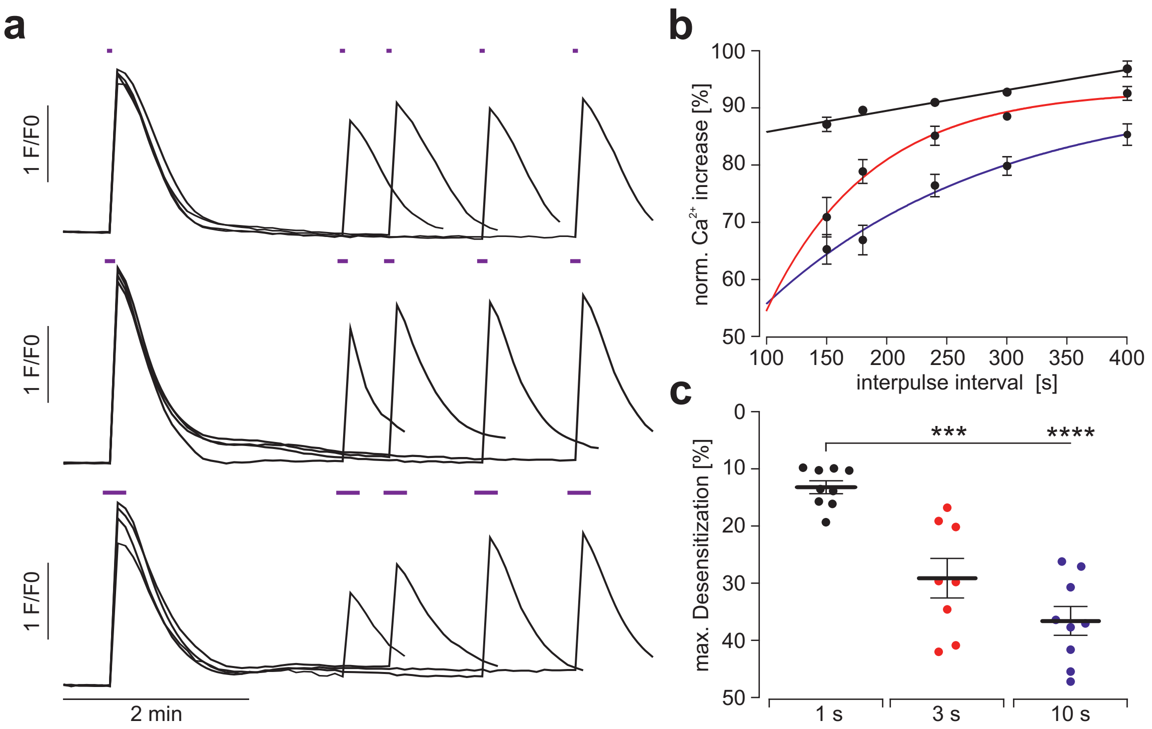

Supplementary Figure 1: Desensitization and recovery from desensitization of light induced Ca^{2+} transients in HEK cells. a, Representative traces of Ca^{2+} transients induced by paired pulses of 1, 3 and 10 s long light pulses (violet bars, 1 mW/mm^2) with increasing interpulse intervals (150, 180, 240, 300 s). **b**, Average normalized Ca^{2+} transient amplitudes in dependence to the interpulse interval and the respective one phase decay fits (1 s: black fit, n = 9; 3 s: red fit, n = 8; 10 s: blue fit, n= 9). **c**, Statistical analysis of the effect of the pulse duration on the maximal desensitization (relative decrease) with an ordinary one-way ANOVA with Tukey's multiple comparison test (p(1 vs. 3 s) < 0.001; p(1 vs. 10 s) < 0.0001; p(3 vs. 10 s) = 0.11). Each dot represents the mean of one coverslip. Data are presented as mean values ± SEM.

Wagdi et al., 2022: Supplementary Figure 2

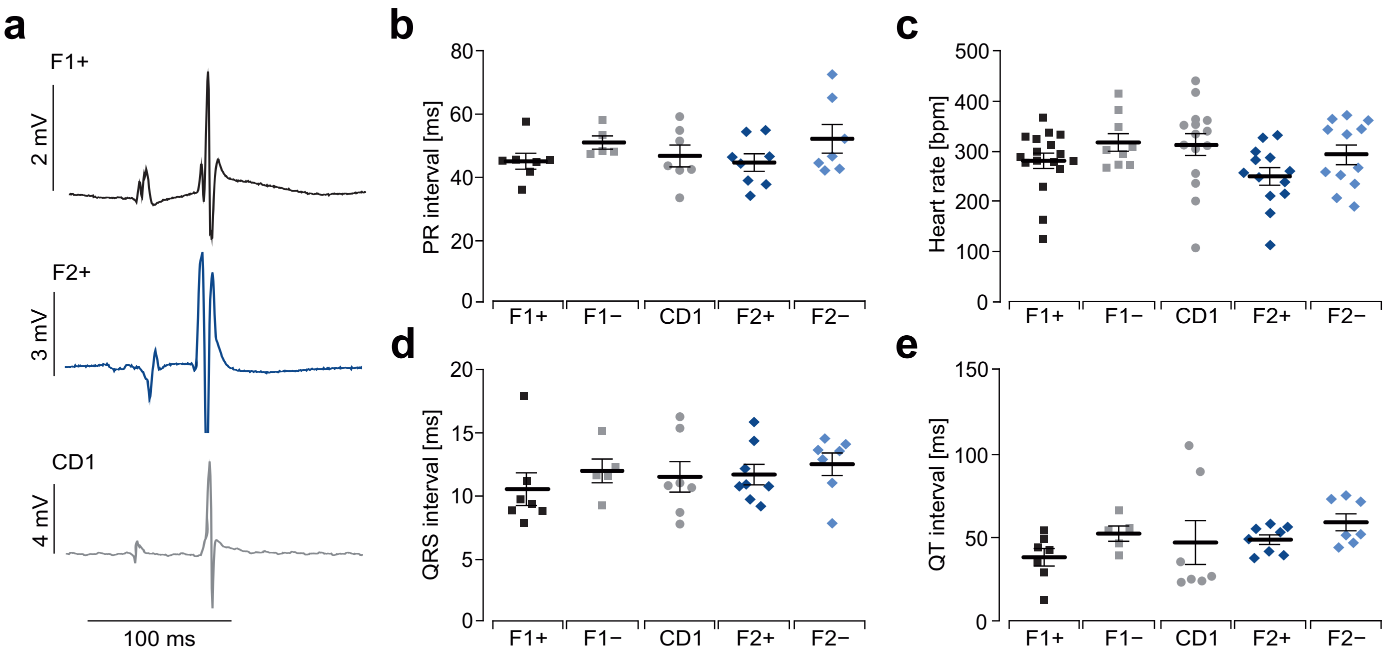

Supplementary Fig. 2: Basal electrocardiogram (ECG) parameters in hOPN5 mice recorded *ex vivo*. a, Representative electrogram recordings from hearts of hOPN5 founder line F1 (black), founder line F2 (blue) and CD1 wild type control mice (gray). **b-e**, Statistical analysis of basal ECG parameters PR interval (b), heart rate (c), QRS interval (d) and QT interval (e) in F1 and F2 hOPN5 expressing hearts (F1+, F2+), from their negative littermates (F1− and F2−) and CD1 wild type controls. To exclude rate dependent effects, only electrogram recordings with a heart rate between 250 and 350 bpm were taken into account in b,d and e. Each dot represents the average from one individual heart. Statistical analysis was performed with ordinary one-way ANOVA test (all p values ≥ 0.096) and Tukey's multiple comparison test (all p values ≥ 0.11). Data are presented as mean values ± SEM.

Wagdi et al., 2022: Supplementary Figure 3

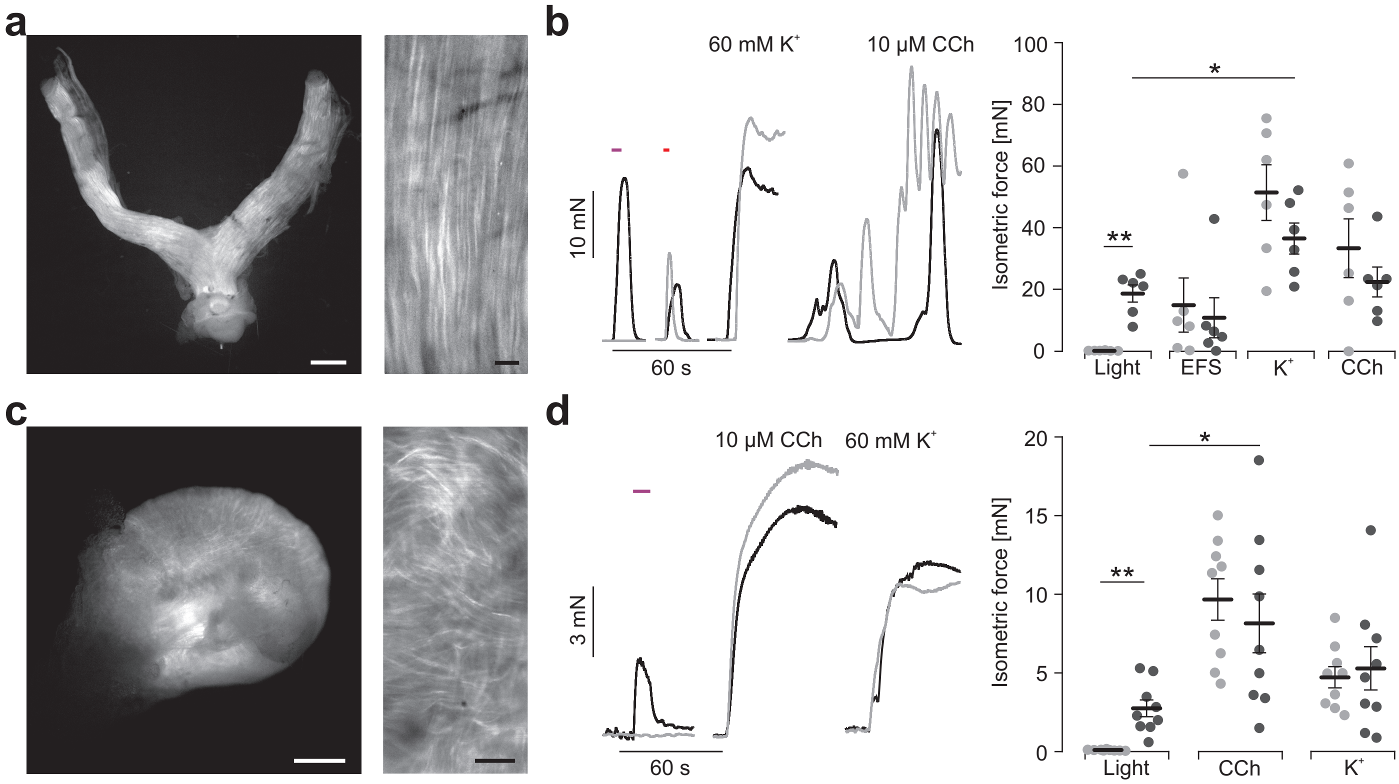

Supplementary Fig. 3: UV light induced contraction in the bladder and uterus of hOPN5 mice. a, eYFP-fluorescence in the whole uterus (left, bar = 2 mm) with magnification (right, bar = 200 µm) of a hOPN5 mouse showing hOPN5/eYFP expression in the myometrium. **b**, Representative traces (left) and aggregated data (right) of isometric force measurements of explanted uterine horns from wildtype controls (N = 3, n = 6; gray) and hOPN5 mice (N = 2, n = 6; black). Contractions were induced by UV light (violet bar, 5 s, 3 mW/mm^2), EFS (red bar, 3 s, 100 Hz, 2 ms, 40 V), 60 mM K^+ and 10 µM CCh. Statistical analysis was performed with 2-way repeated measures ANOVA with Sidak's multiple comparison test between hOPN5 vs WT (p(UV light): 0.0046; p(EFS): 0.53; p(CCh): 0.86; p(K^+): 0.53) and Tukey's multiple comparison test for UV light effects versus different stimuli in hOPN5 samples (p(vs. EFS): 0.75; p(vs. K^+): 0.015; p(vs. CCh): 0.87). **c**, eYFP fluorescence in the whole bladder (left, bar = 1 mm) with magnification (right, bar = 200 µm) of a hOPN5 mouse showing hOPN5/eYFP expression in the tunica muscularis. **d**, Representative traces (left) and aggregated data (right) of isometric force measurements of explanted bladder strips from CD1 wild type controls (gray, N = 3, n = 9) and hOPN5 animals (N = 3, n = 9; in black). Contractions were induced by UV light (violet bar, 10 s, 3 mW/mm^2), 10 µM CCh and 60 mM K^+. Statistical analysis was performed with 2-way repeated measures ANOVA with Sidak's multiple comparison test between hOPN5 vs CD1 wild type (p(UV light): 0.0034; p(K^+): 0.98; p(CCh): 0.93) and Tukey's multiple comparison test for UV light effects versus different stimuli in hOPN5 samples (p(vs. K^+): 0.09; p(vs. CCh): 0.012). Data are presented as mean values ± SEM. *p< 0.05, **p< 0.01.
