# Supplementary material for: Selective optogenetic control of Gq signaling using human Neuropsin
Source: Nat Commun. 2022 Apr 1;13:1765. doi: 10.1038/s41467-022-29265-w (PMC8975936; doi:10.1038/s41467-022-29265-w)
Supplement: Supplementary file 2 — Reporting Summary [file 41467_2022_29265_MOESM2_ESM.pdf]

nature portfolio

Corresponding author(s): Tobias Bruegmann
Philipp Sasse

Last updated by author(s): Feb 10, 2022

Reporting Summary

Nature Portfolio wishes to improve the reproducibility of the work that we publish. This form provides structure for consistency and transparency in reporting. For further information on Nature Portfolio policies, see our Editorial Policies and the Editorial Policy Checklist.

Statistics

For all statistical analyses, confirm that the following items are present in the figure legend, table legend, main text, or Methods section.

n/a	Confirmed	
[ ]	[x]	The exact sample size (*n*) for each experimental group/condition, given as a discrete number and unit of measurement
[ ]	[x]	A statement on whether measurements were taken from distinct samples or whether the same sample was measured repeatedly
[ ]	[x]	The statistical test(s) used AND whether they are one- or two-sided<br>*Only common tests should be described solely by name; describe more complex techniques in the Methods section.*
[x]	[ ]	A description of all covariates tested
[x]	[ ]	A description of any assumptions or corrections, such as tests of normality and adjustment for multiple comparisons
[ ]	[x]	A full description of the statistical parameters including central tendency (e.g. means) or other basic estimates (e.g. regression coefficient) AND variation (e.g. standard deviation) or associated estimates of uncertainty (e.g. confidence intervals)
[ ]	[x]	For null hypothesis testing, the test statistic (e.g. *F*, *t*, *r*) with confidence intervals, effect sizes, degrees of freedom and *P* value noted<br>*Give P values as exact values whenever suitable.*
[x]	[ ]	For Bayesian analysis, information on the choice of priors and Markov chain Monte Carlo settings
[x]	[ ]	For hierarchical and complex designs, identification of the appropriate level for tests and full reporting of outcomes
[x]	[ ]	Estimates of effect sizes (e.g. Cohen's *d*, Pearson's *r*), indicating how they were calculated

Our web collection on statistics for biologists contains articles on many of the points above.

Software and code

Policy information about availability of computer code

Data collection	All commercially available softwares used are clearly stated in the methods. The custom made software for contraction analysis of single cardiomyocytes will be made available on an open source software platform: https://github.com/awagdi0/MOCA/
Data analysis	Statistical data are shown as mean ± standard error of the mean and were analyzed with the GraphPad Prism 8 software. The ecg signal and force measurements were amplified with an animal bio-amplifier (Animal Bio Amp 223 FE136, AD Instruments) and a PowerLab 16/35 recording system and analyzed with LabChart 8.1.16 software.

For manuscripts utilizing custom algorithms or software that are central to the research but not yet described in published literature, software must be made available to editors and reviewers. We strongly encourage code deposition in a community repository (e.g. GitHub). See the Nature Portfolio guidelines for submitting code & software for further information.

Data

Policy information about availability of data

All manuscripts must include a data availability statement. This statement should provide the following information, where applicable:

- Accession codes, unique identifiers, or web links for publicly available datasets
- A description of any restrictions on data availability
- For clinical datasets or third party data, please ensure that the statement adheres to our policy

Data will be provided upon personal and reasonable request to the corresponding authors. Data can only made available upon request since results from the high throughput screen are proprietary to Bayer company. Importantly, we do not have any data which has to be published like sequencing, structures or proteomic/ transcriptomics screens.

Field-specific reporting

Please select the one below that is the best fit for your research. If you are not sure, read the appropriate sections before making your selection.

- [x] Life sciences
- [ ] Behavioural & social sciences
- [ ] Ecological, evolutionary & environmental sciences

For a reference copy of the document with all sections, see nature.com/documents/nr-reporting-summary-flat.pdf

Life sciences study design

All studies must disclose on these points even when the disclosure is negative.

Sample size	We could not determine effect sizes before since these were completely new experiments with non-foreseeable results. Whenever possible we had sample sizes clearly proving very significant p values or high n numbers allowing to exclude any effects by classical statistical tests. All n and p values are given in the legends or the text.
Data exclusions	No data were excluded.
Replication	Experiments were performed in three different labs (WG Sasse, WG Bruegmann, Bayer) and all showed comparable and reproducible results.
Randomization	This is not relevant to our study since phenotypes were obvious due to fluorescence expression and light effects. Furthermore we did not look into effects of treatments within the same kind of cells or groups. Used mice were equally male and female and of various ages.
Blinding	Blinding was not possible due to clear light effects but also not necessary since all analysis was objective with clearly pre-defined criteria.

Reporting for specific materials, systems and methods

We require information from authors about some types of materials, experimental systems and methods used in many studies. Here, indicate whether each material, system or method listed is relevant to your study. If you are not sure if a list item applies to your research, read the appropriate section before selecting a response.

Materials & experimental systems

n/a	Involved in the study	
[ ]	[x]	Antibodies
[ ]	[x]	Eukaryotic cell lines
[x]	[ ]	Palaeontology and archaeology
[ ]	[x]	Animals and other organisms
[x]	[ ]	Human research participants
[x]	[ ]	Clinical data
[x]	[ ]	Dual use research of concern

Methods

n/a	Involved in the study	
[x]	[ ]	ChIP-seq
[x]	[ ]	Flow cytometry
[x]	[ ]	MRI-based neuroimaging

Antibodies

Antibodies used	All antibodies used are clearly described in the methods parts.<br>aActinin: Sigma (A7811, 1:400)<br>asmooth muscle actinin: Sigma-Aldrich (A2547)<br>ßIIITubulin: Biozym/BioLegend (802001, 1:800)<br>cKit: Linaris (MAK5302, 1:400)<br>Oct3/4: SantaCruz Biotechnology (sc-9081, 1:100)<br>HCN4: Alomone (APC-052, 1:200)<br>eGFP: Chromotek (3H9-100, 1:800; 1:400 and 1:100) and Sigma-Aldrich (11.814.460.001, 1:800)<br>All secondary antibodies are clearly identified in the methods section.
Validation	All antibodies used have been often validated and reported before by other scientist. None of the quantitative analysis are depending on antibodies. Relevant citations are: Vogt, Theranostics 2021; Bruegmann, Nature methods 2010; Vogt, Cardiovascular Research 2015, Roell, Nature 2007; Kozasa, Y. et al. (2018) J. Physiol. 596, 809;

Eukaryotic cell lines

Policy information about cell lines

Cell line source(s)	Commercially available, generated by us or given by collaboration partners. Each source is clearly defined and citations are given for the source.<br>HEK cells (AD293, ATCC, U.S.), R1 mouse embryonic stem cells (ES cells)

Authentication	None of the cell lines were additionally authentizised since all are standard cell lines and have not been newly generated for this study. HEK knockout cell lines were functionally tested by positive controls.
Mycoplasma contamination	All cell lines are regularly tested for possible myoplasma contamination. Results were always negative.
Commonly misidentified lines<br>(See ICLAC register)	None.

Animals and other organisms

Policy information about studies involving animals; ARRIVE guidelines recommended for reporting animal research

Laboratory animals	This is how we stated this in the methods: "Mice were kept in 12/12 h dark/light cycles with food and water ad libido at room temperature and standard humidity. [...] hOPN5/eYFP transgenic mice were created by pronuclear injection of FVB/N mice using standard procedures by the core facility of the Max-Planck Institute of Experimental Medicine,Göttingen. [...] Positive founder mice were mated with CD1 wild-type animals to obtain heterozygous offspring and wild type littermate controls. [...] Including all experiments, we used 34 male and 29 female transgenic OPN5 mice<br>203 (16 ± 1.1 weeks old), 31 male and 15 female wild type siblings (12 ± 0.85 weeks old) and 15 male and 18 female CD1 wild type mice (27 ± 2.5 weeks old) were used as controls. [...] Melanopsin expressing transgenic mice were generated using a previously described G4 embryonic stem cell line stably transfected with the CAG-melanopsin-IRES-GFP plasmid. [...] The mice investigated in this study were back-crossed at least 10 generations. Mice of this transgenic mouse line were in total 2 male and 3 female (51 ± 4.2 weeks old) and 5 female CD1 wild type mice (20 ± 1.1 weeks old) as controls."
Wild animals	No wild animals were used in this study.
Field-collected samples	No field collected samples were used in this study.
Ethics oversight	"All animal work conformed to the European Guideline for animal experiments 2010/63/EU and the mouse generation was approved by the Niedersächsische Landesamt für Verbraucherschutz und Lebensmittelsicherheit (approval number 33.9-42502-04-16/2352) and by the Landesamt für Natur, Umwelt und Verbraucherschutz Nordrhein-Westfalen (approval number AZ.84-02.04.2012A146)."

Note that full information on the approval of the study protocol must also be provided in the manuscript.
