# Supplementary material for: Selective optogenetic control of Gq signaling using human Neuropsin
Source: Nat Commun. 2022 Apr 1;13:1765. doi: 10.1038/s41467-022-29265-w (PMC8975936; doi:10.1038/s41467-022-29265-w)
Supplement: Supplementary file 3 — Description of Additional Supplementary Files [file 41467_2022_29265_MOESM3_ESM.pdf]

Title: Supplementary Movie 1:
Description: Representative video of Ca2+ imaging to determine the light sensitivity of hOPN5 in polyclonal HEK cells (corresponding to Figure 2a). XRhod-1-AM signal is shown in fire color code (blue to yellow with increasing fluorescence signal). Cyan dots in the top left corner indicate the five UV light pulses (385 nm, 100 ms) with 0, 0.1, 0.3, 1, 3, 10 µW/mm2, respectively. Video is displayed at 30x speed.

Title: Supplementary Movie 2:
Description: Video showing the expression of hOPN5/eYFP (green) in a 3D rendered volume (normal shading) of light-sheet Z-stack images after tissue clearing. The posterior surface of the heart was cropped to visualize the distribution of hOPN5/eYFP in the endocardium.

Title: Supplementary Movie 3:
Description: 3D rendered volume (blend shading) of light-sheet Z-stack images of cleared hearts with hOPN5/eYFP shown in green and HCN4 staining shown in purple. The posterior wall of the right atrium as well as a rectangular window anterior to the tricuspid valve were cropped to visualize the sinus and atrioventricular nodes.
